# Digital Psychological Wellbeing Interventions for Family Carers of Children and Adults With Intellectual and Developmental Disabilities: A Systematic Review

**DOI:** 10.1111/jar.70081

**Published:** 2025-07-11

**Authors:** Magda M. Apanasionok, Andreas Paris, Joanna Griffin, Richard P. Hastings, Ellie Finch, Debbie Austin, Samantha Flynn

**Affiliations:** ^1^ Centre for Research in Intellectual and Developmental Disabilities (CIDD) University of Warwick Coventry UK; ^2^ School of Social Policy and Society University of Birmingham Birmingham UK

**Keywords:** digital intervention, mental health, online, parents, systematic review, wellbeing

## Abstract

**Background:**

This review explored (1) what digital psychological wellbeing interventions for family carers of people with intellectual and developmental disabilities were reported in the literature, (2) evidence about their effectiveness, (3) factors affecting their implementation and (4) experiences of family carers who attend them.

**Methods:**

Seven databases were searched using search terms related to intellectual and developmental disabilities, carer role, wellbeing and digital delivery formats. Data from 23 studies were synthesised narratively.

**Results:**

Identified interventions were categorised in five broad groups: mind–body, relaxation, mindfulness and acceptance; psychoeducation and support groups; positive thinking and self‐compassion; spiritual; and expressive writing. Only 43% of included studies met seven (100%) or six (85%) quality indicators based on the Mixed Methods Appraisal Tool.

**Conclusions:**

There is a developing literature on digital interventions for family carers of people with intellectual and developmental disabilities. Digital supports will likely become more important with continuing technological advances and increasing need.

**Summary**

- People who care for their family member with intellectual and developmental disabilities can struggle with their mental health.
- Struggling with mental health includes feeling low or anxious.
- We wanted to find out what research has been done about online or virtual ways of helping family carers with their mental health.
- Online help can include websites and resources on the internet.
- Virtual help can include talking with a therapist through the internet.
- We found five different types of therapy that have been used to help family carers with their mental health.
- None of the types of therapy have been tested enough to say they definitely help.
- But some of the therapies are showing promise to be helpful.
- More research is needed to test if those therapies can help family carers with their mental health.

## 1 Introduction

Parents of children or adults with intellectual and developmental disabilities^1^ are often reported as being at increased risk for stress and mental health problems (Masefield et al. 2020; Rydzewska et al. 2021). Their caring role may have a greater impact on their physical health, psychological wellbeing and personal lives than for other carers (Totsika et al. 2017). In addition to being concerning for family carers of people with intellectual and developmental disabilities, poor parental psychological wellbeing may be a risk factor for mental health or behaviour problems in children and adults with intellectual and developmental disabilities (Bailey et al. 2019; Benson 2023; Yorke et al. 2018). There is a pressing and continuous need to provide effective and timely support for family carers of people with intellectual and developmental disabilities, particularly to address the aforementioned challenges of caring for their family member. Access to a diverse and accessible range of interventions is critical in supporting a diverse population of family carers (Williamson and Perkins 2014), including an ageing population of family carers (Mahon et al. 2019).

Despite the clear need for family carers to receive good mental health and wellbeing support, there is a paucity of robust evaluations of interventions that have been developed or adapted for this population (Glidden et al. 2021). Of those that have been tested, promising results have been found for cognitive behavioural therapy (CBT) to improve stress and depression (Da Paz and Wallander 2017; Singer et al. 2007) and mindfulness‐based interventions (e.g., mindfulness‐based stress reduction, acceptance and commitment therapy [ACT]) (Dykens et al. 2014; Lunsky et al. 2017; Yu et al. 2019).

Family carers have reported challenges (e.g., childcare, transport, health problems) to attending regular face‐to‐face interventions (Lunsky et al. 2017). Even before the COVID‐19 pandemic and related restrictions necessitated a move to online (e.g., web‐based platforms or resources) or virtual (e.g., support sessions delivered in real‐time) delivery of interventions, a digital approach seemed to be a promising and flexible alternative to face‐to‐face intervention delivery (Flynn et al. 2020; Lunsky et al. 2021).

There has been one recent systematic review of the literature examining the impact of telehealth broadly for family carers of children with neurodevelopmental disorders (e.g., autism, ADHD, sensory processing disorder, Fragile X syndrome, disruptive behaviours) (Kelson and Dorstyn 2023). Telehealth enables the remote real‐time delivery of healthcare services via devices including a telephone, computer or tablet. This definition of telehealth is akin to our definition of virtual interventions; however, alternative uses of the term telehealth also include online interventions. Attention should therefore be paid to individual definitions of the terminology related to telehealth and digital interventions. The Kelson and Dorstyn review was primarily focused on the quantitative treatment effects of telehealth in this population of family carers and also did not report qualitative data. From the 12 studies included, most of which excluded family carers of children with comorbid conditions including intellectual disabilities, the evidence suggested that telehealth interventions can be used to improve mental health problems in family carers of children with neurodevelopmental disorders and that the methodological quality of the majority of studies included in the review was acceptable. Whilst the Kelson and Dorstyn review provides useful data about the effectiveness of telehealth interventions for family carers of (primarily) children with neurodevelopmental conditions, there is scope for a further review of the literature that more broadly and explicitly focuses on both online and virtual psychological wellbeing interventions for family carers of children and adults with intellectual and developmental disabilities. Further, our review aims to gather both quantitative and qualitative data to answer a series of questions, including those about participants' perceptions of and feedback about the interventions. These data can complement our understanding of the experiences of digital interventions and what factors affect successful implementation to inform future research, practice and policy recommendations.

The current review thus focused on the following research questions: (1) What digital psychological wellbeing interventions for family carers of people with intellectual and developmental disabilities have been reported in the literature?; (2) What is the evidence for their effectiveness in improving family carers' wellbeing?; (3) What are the factors that may affect the implementation of these interventions?; and (4) What are the experiences of digital interventions for family carers of people with intellectual and developmental disabilities?

## 2 Methods

The protocol for the systematic review was co‐authored by family carers of people with intellectual and developmental disabilities and registered with PROSPERO (CRD42023407687) prior to searches commencing. The systematic review is reported in line with PRISMA guidelines (Page et al. 2020) and was conducted with the oversight of an advisory group of family carers of people with intellectual and developmental disabilities, who also co‐authored this paper. See Table S1 for completed PRISMA reporting checklist.

### 2.1 Review Focus and Inclusion Criteria

The review focused on digital (encompassing online and virtual delivery methods) psychological wellbeing interventions for family carers of children and adults with intellectual and developmental disabilities. For the purpose of this review, intellectual and developmental disabilities are defined as learning (as used in UK National Health Service settings)/intellectual disabilities, autism or genetic syndromes associated with these (e.g., Down syndrome). Further, throughout this review, the broad term ‘psychological wellbeing’ will refer to mental health difficulties such as anxiety and depression, stress and positive wellbeing.

#### 2.1.1 Population

The review focused on family (biological, adoptive, step‐parents, foster or kinship) carers with primary parental responsibility for child(ren) or adult(s) with intellectual and developmental disabilities. Adult siblings and grandparents were included if they assumed this caregiving role. Studies were eligible if data were reported for a group in which ≥ 75% of participants met these criteria. There were no exclusion criteria related to how the diagnosis of intellectual and developmental disabilities was confirmed, or the age, or additional diagnoses of the person with intellectual and developmental disabilities.

#### 2.1.2 Interventions

Studies describing digital interventions (online and/or virtual) explicitly stated as targeting improvement in the psychological wellbeing of family caregivers were included. The intervention could deliver information through non‐static web pages and through an online‐based medium where carers are actively engaged in a mobile application (app), webpage platforms, guided digital self‐help programmes or in any information/therapeutic content delivered using any digital means (e.g., text‐based, digital video). The intervention could be delivered synchronously (i.e., in real time) through an implementer or asynchronously (i.e., not in real time) through an automated moderator (e.g., self‐help interventions, pre‐recorded content). Hybrid models whereby there were also some face‐to‐face or telephone sessions were also considered, provided the digital component accounted for ≥ 75% of intervention time. Telephone sessions were only included in this systematic review if they were delivered alongside, or as an element of, a digital intervention. The intervention could be delivered to individual family carers, or whole families, provided a primary objective was to improve the psychological wellbeing of family carers, with clear therapeutic content and ‘intent’ to improve psychological wellbeing. To be considered for inclusion, interventions had to include more than one ‘session’ (i.e., interaction between the participant and therapist outside of study procedures or the participant accessing online content/module as per intervention timetable) and have a clear start and finish.

#### 2.1.3 Comparator/Control

Studies with any control comparison or none were eligible for inclusion.

#### 2.1.4 Study Designs

One of the key objectives of this review was to comprehensively describe the literature base; thus, any quantitative study design (including randomised controlled trials [RCTs], quasi‐experimental comparisons, controlled evaluations, non‐controlled group evaluations, single case experimental design studies) was eligible for inclusion. Qualitative studies were also included, where data were collected through interviews, questionnaires or other data collection methods or tools. Descriptions of digital interventions and their development were also eligible to inform review question 1, even if primary data were not presented or evaluated.

#### 2.1.5 Outcomes

The primary outcome of interest was the psychological wellbeing of family carers. Given the subjective nature and variable measurement of psychological wellbeing, any study reporting outcomes of related constructs was eligible for inclusion. These constructs may have been positively framed (e.g., life satisfaction, quality of life and happiness) or negatively framed (e.g., mental ill‐health, stress and burnout). Factors affecting the implementation of the intervention and the experiences of the parental caregivers taking part in digital interventions were also collated. Outcomes of interest included any quantitative or qualitative data on parental caregivers' experiences. The effects of the intervention were assessed through reported changes in measures of psychological wellbeing outcomes or differences between groups and associated effect sizes and other statistics.

Secondary outcomes of interest included relationship quality (e.g., with the family member with intellectual and developmental disabilities) and other psychological or social outcomes of relevance to the effectiveness of digital interventions (e.g., the wellbeing of the person with intellectual and developmental disabilities).

### 2.2 Search Strategy

Seven databases were searched initially in May and June 2023. They were: MEDLINE (including Pubmed), EMBASE, PsycINFO, Scopus, Web of Science, CINAHL and Applied Social Sciences Index and Abstracts (ASSIA). Four sets of search terms were used related to: (1) intellectual and developmental disabilities, (2) digital delivery, (3) psychological wellbeing outcomes and (4) family carer roles. Terms in each group were separated with ‘OR’, and then the four sets of terms combined with ‘AND’. The search was not restricted by publication date, publication status or language. See Table 1 for an example search string used in Scopus. Search strings for remaining databases can be found in Table S2.

**TABLE 1:** Search terms for Scopus.

Scopus			
“Intellectual* Disab*” OR “Intellectual* Impairment*” OR “Intellectual* Deficien*” OR “Intellectual* Handicap*” OR “Intellectual* Subnormal*” OR “Intellectual* Retard*” OR “Intellectual* Difficult*” OR “Learning Disab*” OR “Learning Impairment*” OR “Learning Deficien*” OR “Learning Handicap*” OR “Learning Subnormal*” OR “Learning Retard*” OR “Learning Difficult*” OR “Mental* Disab*” OR “Mental* Impairment*” OR “Mental* Deficien*” OR “Mental* Handicap*” OR “Mental* Subnormal*” OR “Mental* Retard*” OR “Mental* Difficult*” OR “Developmental Disab*” OR “Developmental Impairment*” OR “Developmental Deficien*” OR “Developmental Handicap*” OR “Developmental Subnormal*” OR “Developmental Retard*” OR “Developmental Difficult*” OR “Down Syndrome” OR Asperger OR Autis* OR “Autism Spectrum Disorder” OR ASD OR “Pervasive Developmental Disorder” OR PDD OR “Smith‐Magenis” OR Rett* OR “Lesch–Nyhan” OR “Prader‐Willi” OR Angelman OR “fragile X” OR “Cri‐du‐chat” OR “Cornelia de Lange” OR “de Lange” OR “Rubinstein‐Taybi” OR velocardiofacial OR DiGeorge OR Down* OR “Fetal alcohol” OR “Overgrowth syndrome” OR Neurodevelopmental	“online intervention*” OR “online treatment*” OR “online therap*” OR program OR “digital intervention*” OR “digital treatment*” OR “digital therap*” OR “digital program mobile intervention*” OR “mobile treatment*” OR “mobile therap*” OR “mobile program” OR virtual OR “smartphone intervention*” OR “smartphone treatment*” OR “smartphone therap*” OR “web‐based intervention*” OR “web‐based treatment*” OR “web based therap*” OR “internet intervention*” OR “internet treatment*” OR “internet therap*” OR “internet‐based” OR “internet based” OR cyber* OR “cyber intervention*” OR “cyber treatment*” OR “cyber therap*” OR mhealth OR ehealth OR mtherap* OR etherap* OR “m‐health” OR “e‐health” OR “m therap*” OR “e‐therap*” OR Tele* OR telehealth OR telemedicine OR “digital health” OR “mobile app” OR app OR “app‐based”	parent* OR carer* OR care* OR “family carer*” OR “family member*” OR mother* OR father* OR guardian* OR sibling* OR adopt* OR “step‐parent*” OR “step parent*” OR “step‐father*” OR “step father*” OR “step‐mother*” OR “step mother*”	wellbeing OR “well‐being” OR “mental health” OR “quality of life” OR QoL OR happiness OR “life satisfaction” OR stress OR burnout OR “burn‐out” OR anxiety OR anxious OR depress* OR “mental* ill*” OR “posttraumatic stress” OR “post‐traumatic stress” OR resilien* OR “parent* coping” OR “care* copying” OR “parent* satisfaction” OR “care* satisfaction” OR “psychologic* flexib*” OR loneliness OR burden OR “distress W/2 psychologic*” OR “distress W/2 mental”

Forward and backward reference searches were conducted on papers included in the review to identify any potential studies that might have been missed during the initial screening. To identify eligible but not yet published research, the corresponding authors of all included articles were contacted and asked whether they had any other potentially eligible research in press or that was otherwise not included. We also reviewed the reference list of a recently published meta‐analysis in a similar area (Kelson and Dorstyn 2023) to identify potentially relevant papers. New articles underwent abstract and, where applicable, full text screening as described below. To make eligibility decisions, Google Translate was used to translate papers in languages in which the research team was not fluent. Searches were re‐run in February 2024 before the synthesis was finalised to identify any papers published since the initial searches were undertaken.

### 2.3 Study Selection

Articles identified through database searches were exported to a reference management software system (EndNote) and MA completed electronic de‐duplication. Initial screening of titles and abstracts of articles retrieved using the search strategy and those from additional sources were screened by MA using Rayyan software (Ouzzani et al. 2016) against inclusion criteria outlined above. At this stage, papers were excluded only if they clearly did not meet the inclusion criteria. Since not all papers reported delivery mode in the abstract, all studies including an intervention seemingly targeting the psychological wellbeing of family carers were taken forward for full text review unless they clearly stated that sessions were delivered in another format. AP independently verified inclusion of 20% of randomly selected records. All disagreements were discussed, and if consensus was not reached, the study was carried forward to the next stage of the study selection. Agreement was calculated by dividing the number of agreements by the number of agreements and disagreements and multiplying by 100%. The agreement rate for initial study selection was 98.29%.

The full texts of all potentially eligible studies were retrieved and independently assessed for eligibility by MA and AP using a bespoke checklist. Reasons for inclusion and exclusion were recorded. Any disagreements between the reviewers were discussed with another member of the research team until agreement was reached. The agreement rate for full text inclusion was 97.73%.

### 2.4 Data Extraction

Data extraction was completed using a bespoke form that also included items from the Template for Intervention Description and Replication (TIDieR) checklist (Hoffmann et al. 2014) to facilitate extraction of information about the interventions themselves. The following information was extracted: authors, publication year, title, country study was conducted in, publication status, reported conflict of interest, reported funding source, research questions or aims, recruitment strategy, intervention name, rationale for the intervention, materials used, intervention procedure, implementer, mode of delivery, location (if applicable), number and frequency of sessions, personalisation and modifications (if applicable), follow up period and adherence and fidelity of implementation. Demographic information for family carers and people with intellectual and developmental disabilities was also extracted whenever possible. For quantitative and mixed‐methods studies, data on outcome measures, analyses used and results including descriptive and inferential statistics were extracted. For qualitative and mixed‐methods studies, brief descriptions of the data collection methods, analysis method and findings were extracted. Information on barriers and facilitators of implementation was extracted wherever reported.

Data extraction was completed by MA. AP reviewed all of the extracted data and noted where there was any missing information that was then extracted.

### 2.5 Quality Appraisal

Given that a wide range of study designs were eligible for inclusion in the review, methodological quality was assessed using the Mixed Methods Appraisal Tool (MMAT; Hong et al. 2018), using the appropriate version of the MMAT (e.g., RCT, qualitative, quantitative descriptive) for each study design. The MMAT consists of two screening questions for all study designs, and then a different set of five questions for qualitative, quantitative randomised controlled trials (RCTs), quantitative non‐randomised controlled trials, quantitative descriptive and mixed methods research. For each screening question, assessors answer ‘yes’, ‘no’ or ‘can't tell’ for a total of seven questions per MMAT. Quality appraisal was conducted independently by MA and SF for all but one study. Due to potential conflict of interest, quality appraisal for Flynn et al. (2020) was completed by MA and AP. Discrepancies were discussed until consensus was reached. Initial agreement was 83.85%.

### 2.6 Data Synthesis

Given the heterogeneity of study designs and outcome measurement in eligible research, a narrative synthesis method was used (Campbell et al. 2020). It was pre‐specified in our PROSPERO protocol that if there were five or more quantitative studies reporting on sufficiently similar interventions using similar methods, or five or more qualitative studies using similar methods about comparable interventions, meta‐analysis or qualitative meta‐synthesis would be conducted. These criteria were not met.

Data synthesis was guided by the review questions. To address review question 1, a TIDieR checklist (Hoffmann et al. 2014) was used to organise information about the interventions in included studies. To address review question 2, key information about the design and results of each study was tabulated and effectiveness evidence was summarised narratively. To address review question 3, any evidence available about factors that affected implementation was summarised. Finally, to address review question 4, key information about family carers' experiences of included interventions and themes and patterns across the studies was described. Based on feedback from the participants and authors of the included studies, as well as reflections of our research team (which includes family carers), we have created a list of recommendations for implementing digital wellbeing interventions with family carers.

### 2.7 Protocol Deviations

There were two deviations from the pre‐registered protocol. Firstly, initial database searches were returning a very high number of irrelevant results. Therefore, one further list of search terms related to psychological wellbeing outcomes was added to improve the specificity of our searches.

Secondly, during data extraction it became apparent that much of the data pertaining to our research question: ‘What are the factors that may affect the implementation of these interventions?’ were drawn from data about participants' experiences of accessing the interventions. Thus, we made a pragmatic decision to combine the presentation of data pertaining to our third and fourth research questions.

### 2.8 Reflexivity Statement

Five of the authors of this manuscript are family carers of people with intellectual and developmental disabilities. Two family carers, who had previous experience of being involved in research studies, were recruited to be part of the Family Carer Advisory Group. Group meetings were held at key stages to: (i) finalise the plan for the review, including refining search terms (ii) consider initial findings and (iii) disseminate the findings.

## 3 Results

### 3.1 Study Selection

Initial database searches returned 22,576 records. An additional 1429 records were identified when searches were re‐run in February 2024. De‐duplication processes removed 9548 records, leaving 14,457 records. Title, abstract and keywords screening led to 14,197 records being removed. Following this initial screening, full texts of 260 records were sought; only one could not be retrieved. MA and AP independently reviewed full texts of 259 records and excluded 240. See Table S3 for a list of records excluded at this stage.

**FIGURE 1:**
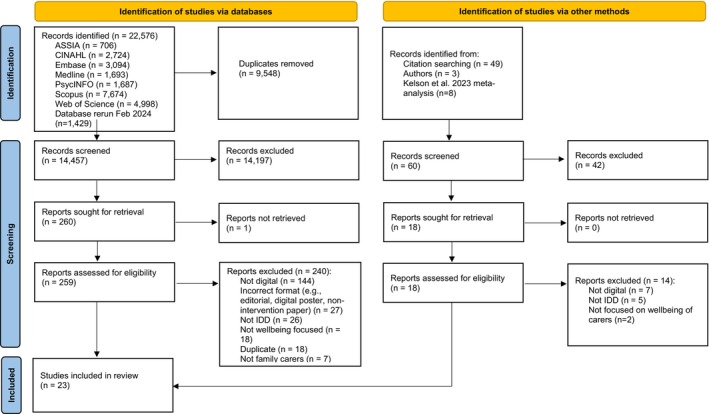
PRISMA 2020 flow diagram.

Through forward and backward reference searches, contact with corresponding authors and a review of the reference list of a recently published meta‐analysis in a similar area (Kelson and Dorstyn 2023), 60 additional potentially relevant records were identified. Following title, abstract and keyword screening, 42 were excluded. Full texts of the remaining 18 records were retrieved and, following review, four were included in the systematic review. See Figure 1 for reasons for exclusion throughout the study selection process. Overall, 23 studies were included in this systematic review.

### 3.2 Study Characteristics

Twenty‐three papers describing 21 different interventions were included in the systematic review. Eighteen of the included papers were published articles, and five were unpublished dissertations. Studies were published or written between 2013 and 2023; five prior to 2020 (when COVID‐19 related restrictions were first imposed globally). Most studies were conducted in the USA (*n* = 13), Canada (*n* = 3) and Australia (*n* = 2). There was one study each from the UK, Saudi Arabia, Peru, India and Türkiye. See Table 2 for details of included studies.

**TABLE 2:** Study descriptions and quantitative outcomes.

Paper ID and country	Study design	Outcomes^a^				Quality ratings
		Timing	Carers	People with intellectual and developmental disabilities	Families	
Ahmed and Raj (2023); *USA*	Feasibility study, single group pre‐post design	Pre‐ and post‐intervention	[+] DASS‐21 [+] WEMWBS [+] SCS			4/7
Bekhet (2017a); *USA*	Feasibility RCT	Pre‐ and post‐intervention (1 week post‐intervention)	[+] PTSS			3/7
Bourke‐Taylor et al. (2022); *Australia*	Single group pre‐post design	Pre‐ and post‐intervention	[+] DASS [+] PGWBI [+] HPAS	[/] CCBS‐2 [+] PedsQL	[/] FES‐CS [+] FES‐FS [/] MyFACE	6/7
Clifford and Minnes (2013); *Canada*	Non‐RCT	Pre‐ and post‐intervention (immediate)	[/] STAI [/] STDS [/] KIPP	[/] SIB‐R [/] SCQ	[/] FSCI	5/7
Curl and Hampton (2023); *USA*	Mixed methods Single group pre‐post design	Pre‐ and post‐intervention	[+] Freiburg Mindfulness Inventory [−] PSI‐SF [/] EIPSES [+] SCS			7/7
Fenning et al. (2023); *USA*	RCT	Pre‐ and post‐intervention (immediate, 6‐ and 12‐months post‐intervention)	[+] FIQ [+] PDH [+] PSI‐4‐SF			3/7
Flujas‐Contreras et al. (2021); *Peru*	Single group pre‐post design	Pre‐ and post‐intervention (3‐months post intervention)	[+] 6‐PAQ [+] AAQ‐II [+] Parenting Stress Scale [+] Emotional Regulation Difficulties Questionnaire [+] Life Satisfaction Scale	[+/−] SDQ		6/7
Flynn et al. (2020); *UK*	Mixed‐ method feasibility RCT	Pre‐ and post‐intervention (12‐week and 6 months post‐randomisation)	[+] HADS [+] WEMWBS [/] PSOC [/] EQ‐5D‐5L	[/] PGS [+/−] Child–Parent Relationship scale	[/] Family APGAR scale [+] Parent‐partner relationship [+] Parent‐partner agreement	7/7
Hemdi and Daley (2017); *Saudi Arabia*	RCT	Pre‐ and post‐intervention (immediate and 8‐weeks)	[+/−] HADS [+] PSI‐SF [+] Arabic Scale of Happiness	[+/−] SDQ [+] ISAA		6/7
Kangavary et al. (2023); *USA*	Case study	Pre‐ and post‐intervention (2‐mid‐point, and 4–6 weeks post‐intervention)	[+] GAD‐7 [+] PSOC [+] PHQ‐9 [+] SHS			3/7
Kuhlthau et al. (2020); *USA*	Feasibility RCT	Pre‐ and post‐intervention (3‐ and 6‐month post‐enrolment)	[+] CAMS‐R [/] VAS [+] CES [+] MOCS‐A [/] PSWQ [+] PHQ‐4 [+] MOS‐SSS [/] PANAS‐P [/] IRI			4/7
Kulbaş and Özabacı (2022); *Turkey*	Mixed‐methods QED	Pre‐ and post‐intervention (immediate and 2‐month post‐intervention)	[+] Psychological Well‐Being Scale [+] SCS [+] Dispositional Hope Scale			7/7
Lake et al. (2022); *Canada*	Single group pre‐post design	Pre‐ and post‐intervention (immediate and 8‐weeks post‐intervention)	[+] WEMWBS [+] Learning and Self‐efficacy (Levels 3 & 4)—competence ratings			7/7
Lunsky et al. (2021); *Canada*	Single group pre‐post design	Pre‐ and post‐intervention (immediate and 3‐months)	[+] DASS‐14 [+] FFMQ [+] BMPS [+] Revised Caregiving Appraisal Scales [+] SCS‐SF	[+] PGS		5/7
Osborn (2021); *Australia*	Feasibility study, single group pre‐post design	Pre‐ and post‐intervention (immediate and 3‐months)	[+] DASS‐21 [+] FFMQ [+] PSI‐4‐SF [+] PHQ‐9			4/7
Padgett (2020); *USA*	RCT	Pre‐ and post‐intervention (immediate)	[/] DASS‐21 [/] MAAS [/] IEM‐P [/] Parental Stress Scale [/] SCS‐SF			2/7
Pandya (2021); *India*	RCT	Pre‐ and post‐intervention	[+] PSI‐SF [+] CAPES [+] MCQ [+] PREQ	[+] CAPES		5/7
Tilson (2022); *USA*	Single group pre‐post design	Pre‐ and post‐intervention (two‐weeks post‐intervention)	[+] AAQ‐II [+] PSI‐4‐SF [+] SC‐SF	[+] VABS‐3—Maladaptive Behaviour Domain		6/7
Whitney and Smith (2015); *USA*	RCT	Pre‐ and post‐intervention (immediate)	[+/−] PSI			3/7
Zhou (2022); *USA*	RCT	Pre‐ and post‐intervention (8‐weeks)	[+] GAD‐7 [+] Perceived Stress Scale [+] MAAS [+] IEM‐P [+] Parental Stress Scale [+] DERS‐SF [/] SWLS [+] SCS‐SF [+] CS [/] BRIEF‐A [+] PHQ‐9	[+] BRIEF‐2 PF		4/7
Zimmerman (2013); *USA*	Mixed‐methods single group pre‐post design	Pre‐ and post‐intervention (immediate)	[/] SIPA	[/] SIPA		7/7

Abbreviations: 6‐PAQ, Parental Acceptance Questionnaire; AAQ‐II, Acceptance and Action Questionnaire; AAQ‐II, Experiential Avoidance Questionnaire‐II; BMPS, Bangor Mindfulness Parenting Scale; BRIEF‐2 PF, Behaviour Rating Inventory of Executive Function—Second Edition: Parent Form; BRIEF‐A, Brief Rating Inventory of Executive Function – Adult Self Report Form; CAMS‐R, Cognitive and Affective Mindfulness Scale‐Revised; CAPES, Child Adjustment and Parent Efficacy Scale; CCBS‐2, Child's Challenging Behaviour Scale—version 2; CES, Current Experience Scale; CS, Compassion Scale; DASS, Depression and Anxiety Stress Scales; DASS‐ 21, Depression Anxiety Stress Scale—21 Items; DASS‐14, Depression Anxiety Stress Scale—14 Items; DERS‐SF, Difficulties in Emotion Regulation Scale—Short Form; EIPSES, Early Intervention Parenting Self‐Efficacy Scale; FES‐CS, Family Environment Scale—Cohesion subscale; FES‐FS, Family Environment Scale—Family and Service Subscale; FFMQ, Five‐Facet Mindfulness Questionnaire; FIQ, Negative Impact scale of the Family Impact Questionnaire; FSCI, Family Stress and Coping Interview; GAD‐7, Generalised Anxiety Disorder 7‐item scale; HADS, Hospital Anxiety and Depression Scale; HPAS, Health Promoting Activities Scale; IEM‐P, Interpersonal Mindfulness in Parenting Scale; IRI, Interpersonal Reactivity Index; ISAA, Indian Scale for Assessment of Autism; KIPP, Kansas Inventory of Parental Perceptions; MAAS, Mindful Attention Awareness Scale; MCQ, Maternal Confidence Questionnaire; MOCS‐A, Measure of Current Status; MOS‐SSS, Medical Outcome Study Social Support Survey; MyFACE, My Family's Accessibility and Community Engagement; Non‐RCT, non‐randomised controlled trial; PANAS‐P, Positive and Negative Affect Schedule‐Positive Subscale; PDH, Intensity subscale of the Parenting Daily Hassles; PedsQL, Paediatric Quality of Life Inventory; PGS, Positive Gains Scale; PGWBI, Psychological General Well‐Being Index; PHQ‐4, Patient Health Questionnaire‐4; PHQ‐9, The Patient Health Questionnaire; PREQ, Parenting Resilience Elements Questionnaire; PSI‐4‐SF, Parental Distress subscale of the Parenting Stress Index‐4, Short Form; PSI‐SF, Parenting Stress Index–Short Form; PSOC, Parenting Sense of Competence Scale; PSWQ, Penn State Worry Questionnaire; PTSS, Positive Thinking Skills Scale; QED, quasi‐experimental design; RCT, randomised controlled trial; SCQ, Social Communication Questionnaire; SCS, Self‐Compassion Scale; SCS‐SF, Self‐Compassion Scale‐Short Form; SDQ, Strengths and Difficulties Questionnaire; SHS, State Hope Scale; SIB‐R, Scales of Independent Behaviour‐Revised Short Form; SIPA, Stress Index for Parents of Adolescents; STAI, State Trait Anxiety Inventory; STDS, State–Trait Depression Scales; SWLS, Satisfaction with Life Scale; VABS‐3, Vineland Adaptive Behaviour Scale; VAS, Visual Analogue Scale; WEMWBS, Warwick‐Edinburgh Mental Well‐Being Scale.

^a^ A full list of outcome measures, with accompanying author information, can be found in the Supporting Information; [+] indicates a significant/meaningful improvement in the outcome of interest in the intervention groups; [−] indicates a significant/meaningful deterioration in the outcome of interest in the intervention groups; [/] indicates no change in the outcome of interest in the intervention groups; [+/−] indicates mixed findings in the outcome of interest in the intervention groups.

#### 3.2.1 Design

RCT designs were used in 10 studies. Four studies aimed to explore acceptability and feasibility, while six studies the primary stated interest was effectiveness or efficacy. Control conditions included treatment as usual, alternative interventions or waitlist. Eight of the included studies used single group pre–post‐test designs. Designs of the remaining studies were: mixed methods study (*n* = 2), a non‐randomised controlled trial (*n* = 1; Clifford and Minnes 2013), a descriptive study (*n* = 1; Luberto et al. 2021) and a case study (*n* = 1; Kangavary et al. 2023).

#### 3.2.2 Participants

In total, 1118 family carers participated in the included studies. The majority of studies included only parents—mothers and fathers (*n* = 9) or only mothers (*n* = 8). Four studies included other family carers (e.g., grandparents, aunts and adult siblings) as well as parents. Two studies did not describe the relationship of the family carers to the person with intellectual and developmental disabilities in sufficient detail.

Most studies (*n* = 16) included family carers of children and adolescents up to 18 years of age. Five studies included family carers of children and adults, while one study recruited only family carers of adults (Lake et al. 2022). One study did not report the age range of people with intellectual and developmental disabilities (Kulbaş and Özabacı 2022).

Thirteen studies recruited family carers of autistic people, while nine studies focused on family carers of people with mixed diagnoses of intellectual and developmental disabilities. One study recruited only family carers of people with intellectual disabilities (Kulbaş and Özabacı 2022). See Table 3 for details of included studies.

**TABLE 3:** Participant descriptions.

	Carers				People with intellectual and developmental disabilities				
Paper ID and country	N	Sex and relationship to person with IDD	Age	Ethnicity	N	Sex	Age (in years unless specified)	IDD Diagnoses	Ethnicity
(Ahmed and Raj 2023; *USA*)	50	96% female (mothers); 4% male (fathers)	M = 42.1 (SD = 7.9) Range = 25–62	84% white; 6% black/African American; 6% Asian American or Latino	NR	NR	NR	44% Down syndrome; 42% autism; 44% ADHD; 22% IDD; 4% GDD; 16% other	NR
(Bekhet 2017a, 2017b; *USA*)	64 (intervention group = 28; control group = 36)	94% female (mothers); 6% male (fathers)	M = 37.5 (SD = 7) Range = 24–61	83% Caucasian; 17% African American, Hispanic, or other	NR	78% male	M = 7.6 (SD = 3.7) Range = 2–17	100% autism	78.1% Caucasian; 21.9% African American, Hispanic, or other
(Bourke‐Taylor et al. 2022; *Australia*)	71	100% female (mothers)	M = 43 (SD = 7)	NR	NR	70% male; 30% female	M = 10.6 (SD = 6.1)	62% autism; 15.5% Cerebral Palsy; 27% intellectual disabilities; 7.7% developmental disabilities; 9.2% Down syndrome.	NR
(Clifford and Minnes 2013; *Canada*)	43 (intervention group = 20; control group = 23)	Intervention group: 100% female (mothers) Control group: 91% female (mothers); 9% male (fathers)	Intervention group: M = 43 (SD: 5.61) Range = 33–53; Control group: M = 43 (SD = 8.42) Range = 26–65	NR	45 (intervention group = 20; control group = 25)	Intervention group: 85% male; 15% female Control group: 96% male; 4% female	Intervention group: M = 9 (SD = 4.83) Range = 2–22 Control group: M = 10 (SD = 4.14) Range = 3–17	Intervention group: 100% autism (4% also had intellectual disabilities) Control group: 100% autism (8% also had intellectual disabilities)	NR
(Curl and Hampton 2023; *USA*)	10	100% female (mothers)	Range = 34–54	11% Asian; 11% black; 22% white; 56% Hispanic	NR	NR	Range = 3–8	100% autism	NR
(Fenning et al. 2023; *USA*)	51 (intervention group = 27; control group = 24)	91% female (mothers)	M = 34.6 (SD = 7.5)	51% Latinx	51 (intervention group = 27; control group = 24)	80% male	M = 52.5 months (SD = 11)	100% autism (68% also had intellectual disabilities)	Majority Latinx
(Flujas‐Contreras et al. 2021; *Peru*)	9	77% female (mothers); 23% male (fathers)	M = 36.11 (SD = 4.88)	NR	NR	85% male; 15% female	M = 3.8 (SD = 2.03)	100% autism (two children also had ADHD)	NR
(Flynn et al. 2020; *UK*)	60 (Be Mindful group = 30; Be Mindful+ group = 30)	92% female (90% mothers; 2% other); 8% male (fathers)	M = 46.09 (SD = 7.71) Range = 33–62	80% White British; 10% Asian/Asian British; 7% white other; 1.5% Black British; 1.5% mixed race	60	55% male; 45% female	M = 13.73 (SD = 8.97) Range = 1–55	90% intellectual disability; 68% autism (some co‐morbidities)	NR
(Hemdi and Daley 2017; *Saudi Arabia*)	62 (intervention group = 32; control group = 30)	100% female (mothers)	Intervention group: M = 32.90 (SD = 7.26) Control group: M = 34.43 (SD = 6.65)	NR	62	NR	Intervention group: M = 63.18 months (SD = 13.68) Control group: M = 58.73 months (SD = 14.07)	100% autism	NR
(Kangavary et al. 2023; *USA*)	1	Female (mother)	45	NR	1	Male	10	Autism	NR
(Kuhlthau et al. 2020; *USA*)	51 (intervention group = 25; control group = 26)	96% female (mothers); 4% male (fathers)	M = 45 (SD = 7.6)	84.3% white; 5.9% black; 3.9% Asian; 2% Hawaiian; 3.9% other	51 (intervention group = 25; control group = 26)	NR	2–11 (51%); 12–16 (31.4%); 17+ (17.6%)	100% autism	NR
(Kulbaş and Özabacı 2022;*Türkiye*)	35 (intervention group = 12; placebo group = 11; control group = 12)	NR	NR	NR	NR	NR	NR	100% intellectual disabilities	NR
(Lake et al. 2022; *Canada*)	126	92.1% female (74.6% mothers; 7.9% sisters; 9.6% other); 7.9% male (5.6% fathers, 1.6% brothers; 0.7% other)	M = 55.8 (SD = 11.3)	57.9% White North American; 0.8% East Asian; 4.8% South Asian; 1.6% South East Asian; 1.6% Black North American; 0.8% Black Caribbean; 0.8% Indigenous; 0.8% Latin American; 23% White European; 1.6% mixed heritage	NR	NR	M = 29 (SD = 10.6)	100% intellectual and developmental disability	NR
(Luberto et al. 2021; *USA*)	51	95% female (mothers); 5% male (fathers)	M = 44.97 (SD = 7.21)	92% white; 3% black; 3% Asian; 2% other	NR	NR	M = 2.10 (SD = 2.69)	100% autism	NR
(Lunsky et al. 2021; *Canada*)	39	89.7% female	M = 52.68 (SD = 6.87) Range = 36–67	NR	40	25% female	M = 20.92 (SD = 5.32) Range = 16–39	100% autism	NR
(Osborn 2021; *Australia*)	12	100% female (mothers)	NR	NR	NR	NR	NR	70% autism	NR
(Padgett 2020; *USA*)	32 (intervention group = 10; control group = 22)	94% female (mothers)	M = 39.03 (SD = 6.48) Range = 29–53	85% white; 6% Hispanic or Latinx; 3% Asian; 3% Native Hawaiian or Pacific Islander; 3% Multiracial	32	NR	M = 7.90 (SD = 2.05)	100% autism	NR
(Pandya 2021; *India*)	137 (intervention group = 79; control group = 58)	100% female (mothers)	Intervention group: M = 35.06 (SD = 1.93) Control group: M = 34.57 (SD = 2.31)	NR	NR	NR	Intervention group: M = 5.78 (SD = 1.67) Control group: M = 5.67 (SD = 1.71)	100% autism	NR
(Tilson 2022; *USA*)	5	100% female (mothers)	NR	80% white; 20% black	5	80% female; 20% male	Range = 2–21	80% Prader Willi Syndrome; 20% autism	NR
(Whitney and Smith 2015; *USA*)	120 (intervention group = 56; control group = 64)	100% female (mothers)	M = 41 Range = 31–50	91% Caucasian	NR	72% male	Range = 3–18	47.9% autism; 15.4% SPD; 4.3% Asperger's syndrome; 3.4% ADHD/ADD; 28.2% other	NR
(Zhou 2022; *USA*)	83 (intervention group = 43; control group = 40)	80% female (67% mothers)	M = 42.97 Range = 30–55	73% white	55 (does not include children whose carers withdrew)	73% male	M = 10.18 Range = 5–18	100% autism	71% white
(Zimmerman 2013; *USA*)	12	100% female (92% mothers; 8% aunt)	M = 43.75	NR	11 (does not include children whose carers withdrew)	18% female; 82% male	M = 15.72	100% autism	NR

Abbreviations: ADD, Attention Deficit Disorder; ADHD, Attention Deficit Hyperactivity Disorder; GDD, Global Developmental Delay; IDD, intellectual and developmental disabilities; NR, not reported; SPD, Sensory Processing Disorder.

### 3.3 Intervention Characteristics

Included studies described 21 different interventions that were categorised in five broad groups: mind–body, relaxation, mindfulness and acceptance (*n* = 9; Fenning et al. 2023; Flujas‐Contreras et al. 2021; Flynn et al. 2020; Kuhlthau et al. 2020; Luberto et al. 2021; Lunsky et al. 2021; Osborn 2021; Padgett 2020; Tilson 2022; Zhou 2022); psychoeducation and support groups (*n* = 6; Bourke‐Taylor et al. 2022; Clifford and Minnes 2013; Hemdi and Daley 2017; Kangavary et al. 2023; Lake et al. 2022; Zimmerman 2013); positive thinking and self‐compassion (*n* = 4; Ahmed and Raj 2023; Bekhet 2017a, 2017b; Curl and Hampton 2023; Kulbaş and Özabacı 2022); spiritual (*n* = 1; Pandya 2021); and expressive writing (*n* = 1; Whitney and Smith 2015). See Table 4 for intervention characteristics.

**TABLE 4:** Intervention descriptions.

Paper ID and country	Intervention	Synchronous/Asynchronous	Implementer	Mode of delivery	Frequency	Description	Theoretical underpinnings
(Ahmed and Raj 2023; *USA*)	Web‐based self‐compassion intervention	Asynchronous	None	Online modules on secure website	Four weekly sessions (~12 min each) Two reminders each week (if needed)	Psychoeducation and written experimental activities. Topics included: self‐compassion, self‐kindness, common humanity and mindfulness.	Transactional Model (Hastings 2002)
(Bekhet 2017a, 2017b; *USA*)	Positive thinking training	Asynchronous	None	Pre‐recorded PowerPoint presentations with a voiceover	Six weekly sessions	First session was an introduction to positive thinking and content of the remaining sessions. Topics for remaining sessions included: transforming negative thoughts into positive thoughts; finding positive aspects; interrupting and challenging pessimistic thoughts; breaking problems down; practicing positive thinking; controlling negative thoughts. Participants were asked to complete homework in‐between sessions to practice techniques.	Not reported
(Bourke‐Taylor et al. 2022; *Australia*)	The Healthy Mothers Healthy Families (HMHF)	Synchronous Asynchronous	Occupational Therapist, trainees, expert guests (e.g., general practitioner, physiotherapist, counsellor, dietician) None	Group face‐to‐face session Online modules on a website	One 1‐day workshop at the beginning of the intervention Six sessions completed at participants' pace.	Topics included: The Journey of Mothers; Health and Research Findings; What Mothers Say About Stress; Healthy Mind, Healthy Mother; Active Healthy Mother; Managing and staying strong. Participants also had access to a workbook.	Not reported
(Clifford and Minnes 2013; *Canada*)	Online Support Group for Parents of Children with Autism Spectrum Disorders	Synchronous	Clinician	Online group chat using pseudonyms	Seven or eight 1‐h weekly or bi‐weekly chats	Sessions were guided by participant‐led decisions (e.g., frequency, times, topics of meetings) and iterative participant feedback was used to develop future sessions. Topics were: treatment issues, the impact of autism on families, managing behaviour problems, coping with stress, advocacy, dealing with schools and the community, useful resources and transitions. Topics varied between groups, depending on the interests of group members.	Stress Buffering Model (Cohen and Wills 1985)
(Curl and Hampton 2023; *USA*)	Mindful Self‐Compassion (MSC) workshop	Synchronous Asynchronous (optional)	Mindful Self‐Compassion coach None	Group video‐conferencing Recordings of sessions for participants who could not attend	Three weekly 75‐min sessions Released after each session	Each session included a meditation, brief exercise and Mindful Self‐Compassion technique. Participants also had access to a workbook. Participants were asked to complete homework in‐between sessions.	Not reported
(Fenning et al. 2023; *USA*)	Mindfulness‐Based Stress Reduction (MBSR)	Synchronous	Mindfulness instructor and doctoral students	Group video‐conferencing Plus a pre‐intervention digital orientation session Online meditation retreat	Eight weekly 2‐h sessions One 6‐h retreat after session six	The weekly sessions included mindfulness exercise, as well as small and whole group discussions. Participants were asked to practice mindfulness daily at home for up to 45 min, guided by audio recordings and a workbook.	Not reported
(Flujas‐Contreras et al. 2021; *Peru*)	The Forest of Parenting	Synchronous	Therapists	Individual video‐conferencing	Eight weekly 1‐h sessions	Sessions focused on: introduction to the intervention, emotional acceptance and perspective taking, psychological flexibility, emotional regulation strategies, cognitive distancing skills, generalisation of skills, functional analysis, promoting awareness, adaptive and positive parenting strategies and behavioural management strategies.	Not reported
(Flynn et al. 2020; *UK*)	Be Mindful	Synchronous (Be Mindful + only) Asynchronous	Peer mentors Mindfulness trainers	Individual telephone mentoring calls Online modules on a website	Three bi‐weekly 30‐min sessions timed to coincide with typical key Be Mindful completion milestones Ten sessions completed across four or more weeks, at participants' own pace	Manualised and based on the GROW [Goals, Reality, Options, Way Forward] Model (Whitmore 1996). The aim was to encourage and motivate participants to start and complete Be Mindful. Sessions included pre‐recorded audio and video instructions based on elements of MBCT. Participants were asked to complete 12 assignments outside of sessions, had access to six course handouts and received motivational emails throughout the interventions.	Not reported
(Hemdi and Daley 2017; *Saudi Arabia*)	Psychoeducation Intervention delivered via WhatsApp for mothers of children with Autism Spectrum Disorder	Synchronous	Therapists	Face‐to‐face session WhatsApp sessions	One 1‐h session Four 30‐min sessions; frequency not reported	Sessions covered: aetiology of autism, stress, behaviour problems and available resources and support. Participants also had access to an intervention booklet about the sessions.	Not reported
(Kangavary et al. 2023; *USA*)	Coping Options for Parent Empowerment (Project COPE)	Synchronous	Not reported	Telehealth group sessions	Four sessions; frequency and length not reported	Sessions focused on coping strategies, including: emotional awareness, strategic attention, mindfulness, valued action, emotion modelling, catastrophic thinking and independence. The intervention was available in English and Spanish. Sessions were scheduled in the after work/school hours.	Not reported
(Kuhlthau et al. 2020; Luberto et al. 2021; *USA*)	Stress Management and Resiliency Training‐Relaxation Response Resiliency Program (SMART‐3RP)	Synchronous	Clinical Psychologist	Group video‐conferencing	Eight weekly sessions	Sessions focused on training in three areas—mind–body skills, cognitive‐behavioural skills and positive psychology skills. Each session had a theme and included did active teaching, skills practice and group discussions. Participants were asked to practice learnt skills in between sessions.	Not reported
Kulbaş and Özabacı 2022; *Türkiye*)	Positive Psychology‐Based Online Group Counselling Program	Synchronous	Researcher	Group video‐conferencing	Ten weekly 60–90‐min sessions	Topics included: introduction to positive psychology, identifying strengths, self‐acceptance, getting to know feelings, self‐compassion, relationships with others, thanksgiving, optimism and hopeful life.	6‐Dimensional Model of Psychological Well‐being (Ryff 1989)
(Lake et al. 2022; *Canada*)	Extension for Community Healthcare Outcomes (ECHO)	Synchronous	Two mothers of adults with intellectual disabilities, two psychologists, two operations staff; expert guests	Group video‐conferencing	Six weekly 90‐min sessions Plus a pre‐intervention digital orientation session	Each session covered COVID‐19 news updates, a wellness activity, didactic teaching and time for networking. Topics of the sessions were: health care communication, mental health assessment and treatment, grief and loss, health care planning, decision making and caregiver mental health. Participants also had access to a website containing slides from the sessions, links, resources and participants comments from sessions.	Not reported
(Lunsky et al. 2021; *Canada*)	Group Virtual Mindfulness‐Based Intervention for Parents of Autistic Adolescents and Adults	Synchronous	Clinicians and parent advisors	Group video‐conferencing	Six weekly 90‐min sessions Plus a pre‐intervention digital orientation session	Each session included homework review, brief meditation, introduction to a new meditation technique and group discussion. Participants were asked to do mindfulness exercises in‐between sessions. Practices were adapted for older people, and those who may have had sensory or mobility issues.	Not reported
(Osborn 2021; *Australia*)	Learning mindfulness	Asynchronous	None Researcher	Website Standardised emails	Two weekly modules Four emails across 2 weeks	The website was designed specifically for the research study and contained general mindfulness information, two modules about using mindfulness in every day life and activities, and a frequently asked questions section. Daily 10‐min mindfulness practice audio recordings were provided in male and female voices. Email content included additional mindfulness tips.	Double ABCX Model (McCubbin and Patterson 1983); Family Systems Theory (Cox and Paley 1997)
(Padgett 2020; *USA*)	Mindful Parenting	Asynchronous	None	Qualtrics	Six weekly 1‐h sessions	Sessions included written information, mindfulness practices and homework. Participants were asked questions during the sessions to check their understanding and engagement.	Bluth et al. Model (Bluth et al. 2013)
(Pandya 2021; *India*)	The WhatsApp‐based spiritual posts intervention	Asynchronous	Two spiritual trainers and two rehab social workers	WhatsApp messages in a private group	Fifty weekly posts	Posts were focused on practical use of spirituality as a supportive factor for carers of autistic children. Topics were: focussing on the present moment, acceptance, meditation, relational consciousness, being mindful about negative emotions. The schedule of posts was adjusted to fit with participants' schedules.	Double ABCX Model (McCubbin and Patterson 1983)
(Tilson 2022; *USA*)	Acceptance and commitment therapy	Synchronous Asynchronous	Researcher None	Individual video‐conferencing Recordings on Vimeo	One initial interview Six weekly check‐in sessions Six 5–10‐min videos, sent weekly	Participants had initial interview to identify treatment goals, followed by weekly check‐in sessions. Topics included: identification of values, present moment awareness, diffusion, the matrix, committed action and self‐care.	Coercion Theory (Patterson 2016)
(Whitney and Smith 2015; *USA*)	Emotional Disclosure Through Journal Writing	Asynchronous	None	Reminders to use online Dropbox	Eight weekly prompts	Participants were asked to take no longer than 15 min to journal each time. Journaling prompts were provided to participants according to a structured protocol. There were no restrictions on number of journaling sessions participants could complete each week. Journal entries were submitted via a secure online Dropbox.	Paradigm of Salutogenesis (Antonovsky 1996); Cognitive Theory of Stress and Coping (Lazarus and Folkman 1984)
(Zhou 2022; *USA*)	Mindful Parenting	Synchronous	Researcher	Group video‐conferencing	Eight weekly 90–120‐min sessions Plus a pre‐intervention digital orientation session One follow‐up session 2‐months post intervention	Sessions focussed on different themes of mindful parenting and included didactics and interactive exercises. Participants also had access to a workbook. Participants were asked to practice daily at home for up to 30 min and received reminders via text messages and emails. They had access to videos of guided meditations on YouTube and a mobile app (Insight Timer).	Not reported
(Zimmerman 2013; *USA*)	Web‐Based Groups for Parents of Adolescents with Autism Spectrum Disorders	Synchronous	Graduate student	Group video‐conferencing	Four weekly sessions Plus a pre‐intervention digital orientation session	Sessions focussed on stress reduction, with topics related to parental self‐care, education and puberty, as well as transition to adulthood.	Not reported

Eight studies described theoretical frameworks underpinning their interventions, including the Transactional Model (Hastings 2002), Coercion Theory (Patterson 2016), Paradigm of Salutogenesis (Antonovsky 1996), Cognitive Theory of Stress and Coping (Lazarus and Folkman 1984), Stress Buffering Model (Cohen and Wills 1985), Double ABCX Model (McCubbin and Patterson 1983), 6‐Dimensional Model of Psychological Well‐being (Ryff 1989), Family Systems Theory (Cox and Paley 1997) and the Bluth et al. Model (Bluth et al. 2013). Apart from the Double ABCX Model (McCubbin and Patterson 1983) which was mentioned in two studies, no other framework was referred to more than once, suggesting significant variation in the theoretical underpinnings of the included interventions. Details about theoretical underpinnings for specific interventions are included in Table 4.

Ten interventions were delivered using videoconferencing, seven using online modules, Dropbox or a website and three used online chat systems. Details on how the intervention was delivered were not provided for one intervention (Kangavary et al. 2023). In addition to digital delivery, two interventions included a face‐to‐face element (Bourke‐Taylor et al. 2022; Hemdi and Daley 2017) and one included additional support delivered by family carer peer mentors over the telephone (Flynn et al. 2020). Interventions were delivered by a clinician, trainer or researcher (*n* = 12) or had no implementer (*n* = 5). Three interventions were co‐delivered by peer mentors/family carers and clinicians/researchers (Flynn et al. 2020; Lake et al. 2022; Lunsky et al. 2021). The implementor was not reported for one intervention (Kangavary et al. 2023).

Interventions included between three and 50 sessions. Most interventions had six (*n* = 5), eight (*n* = 5) or ten sessions (*n* = 2). One intervention also included an online mindfulness retreat (Fenning et al. 2023). Sessions were scheduled weekly for most interventions (*n* = 16). Five interventions had varied or no predetermined session frequency or did not report this information.

Twelve interventions were delivered synchronously, six asynchronously, and three used a combination of synchronous and asynchronous delivery methods. Of the interventions delivered synchronously or with a synchronous element, eleven were in group format, three were delivered individually and one did not report the format (Hemdi and Daley 2017).

#### 3.3.1 Rationale for Interventions

Most interventions aimed to reduce the risk of mental ill health and improve wellbeing among family carers of people with intellectual and developmental disabilities. Some studies (post‐2020) additionally mentioned restrictions related to the COVID‐19 pandemic as motivation to work on digitally delivered interventions.

#### 3.3.2 Quality of Reporting

The TIDieR checklist (Hoffmann et al. 2014) was used to guide the data extraction process and help evaluate the quality of reporting of the included interventions. The TIDieR checklist contains 10 broad categories: (1) brief name of the intervention, (2) rationale of the intervention, (3) what was done (including materials and the procedure), (4) who provided the intervention, (5) how the intervention was delivered, (6) where the intervention was delivered, (7) when and how much of the intervention was provided, (8) tailoring, (9) modifications and (10) how well the intervention was delivered. All studies described the following elements in sufficient detail: name of the intervention, rationale, procedures and implementor (where applicable). The following items were most commonly missed or described in insufficient detail in the included papers: tailoring and modifications (*n* = 17), how well the intervention was delivered (*n* = 11) and the materials used (*n* = 8).

### 3.4 Psychological Wellbeing Related Outcomes and Effectiveness

#### 3.4.1 Mind–Body, Relaxation, Mindfulness and Acceptance

Ten studies evaluated mind–body, relaxation, mindfulness or acceptance interventions. Five studies in this group conducted RCTs, two of which focused on the feasibility and acceptability of interventions (Flynn et al. 2020; Kuhlthau et al. 2020) and three focused on their effectiveness or efficacy (Fenning et al. 2023; Padgett 2020; Zhou 2022). Control conditions included waitlist control (*n* = 3) and alternative interventions (*n* = 2; psychoeducational support and target intervention with additional support). One RCT met all quality indicators on the MMAT (Hong et al. 2018; see Table 2 and Table S5 for quality rating for all included studies), two met four, one met three, while one met only two quality indicators (from seven). Four studies used single group pre‐post designs (Flujas‐Contreras et al. 2021; Lunsky et al. 2021; Osborn 2021; Tilson 2022). Two studies met six quality indicators on the MMAT, one five and one four. Luberto et al. (2021) conducted a descriptive study and met six quality indicators on the MMAT.

All 10 studies reported mostly positive findings in terms of family carer psychological wellbeing being improved. Improvements were reported for outcomes including: stress/distress (Fenning et al. 2023; Flujas‐Contreras et al. 2021; Osborn 2021; Tilson 2022; Zhou 2022), emotional regulation (Flujas‐Contreras et al. 2021; Zhou 2022), life satisfaction (Flujas‐Contreras et al. 2021), anxiety and depression symptoms (Flynn et al. 2020; Osborn 2021; Zhou 2022), wellbeing (Flynn et al. 2020; Osborn 2021), mindfulness (Kuhlthau et al. 2020; Lunsky et al. 2021) and self‐compassion (Lunsky et al. 2021; Tilson 2022). Four studies (Kuhlthau et al. 2020; Padgett 2020; Tilson 2022; Zhou 2022) also reported no significant changes on some outcomes of interest (distress, self‐compassion, stress and life satisfaction) at post‐test compared to baseline.

Of interest may also be that Flynn et al. found that family carers who received peer support in addition to the Be Mindful intervention experienced greater improvement in measures related to psychological wellbeing at post‐test than the group which had access to the Be Mindful intervention without peer support, but this change was not statistically significant. Additionally, two studies (Fenning et al. 2023; Lunsky et al. 2021) reported no differences between virtual and in‐person delivery of their interventions.

#### 3.4.2 Psychoeducation and Support Groups

Six studies evaluated psychoeducation interventions and support groups. Hemdi and Daley (2017) conducted the only RCT in this group, which met six (of seven) quality indicators on the MMAT. The study focused on the efficacy of the intervention and included care as usual as the control condition. One study (Clifford and Minnes 2013) used a non‐randomised controlled trial design and met five quality indicators on the MMAT. Bourke‐Taylor et al. (2022) and Lake et al. (2022) both used single group pre‐post designs. One met all quality indicators on the MMAT (Hong et al. 2018) and the other six. Zimmerman (2013) used mixed‐methods and met all quality indicators on the MMAT. Kangavary et al. (2023) described a case study and met three quality indicators on the MMAT.

Four of the studies reported mostly positive effects of the interventions on the psychological wellbeing of family carers. Improvements were reported for outcomes including: stress/distress (Hemdi and Daley 2017), happiness (Hemdi and Daley 2017), anxiety and depression symptoms (Bourke‐Taylor et al. 2022; Kangavary et al. 2023), wellbeing (Bourke‐Taylor et al. 2022; Lake et al. 2022) and hope (Kangavary et al. 2023). Hemdi and Daley also reported no significant change on one of the measures of interest (stress at post‐test when comparing the intervention and control groups). Two studies (Clifford and Minnes 2013; Zimmerman 2013) found no changes on any measures related to family carer psychological wellbeing.

#### 3.4.3 Positive Thinking and Self‐Compassion Therapy

Five studies evaluated interventions based on positive thinking or self‐compassion therapy. Two of the studies (Bekhet 2017a, 2017b) were RCTs and met three one (out of seven) quality indicators on the MMAT. Both RCTs focus on acceptability, fidelity and feasibility and included care as usual as their control condition. Two studies used single‐group pre‐post designs (Ahmed and Raj 2023; Curl and Hampton 2023). One met all quality indicators on the MMAT (Curl and Hampton 2023) while the other met four. Kulbaş and Özabacı (2022) used a mixed‐methods design and met all quality indicators on the MMAT.

All studies reported mostly positive results of the interventions on the psychological wellbeing of family carers. Improvements were reported for outcomes including: anxiety and depression symptoms (Ahmed and Raj 2023), wellbeing (Ahmed and Raj 2023; Kulbaş and Özabacı 2022), self‐compassion (Ahmed and Raj 2023; Curl and Hampton 2023; Kulbaş and Özabacı 2022), positive thinking (Bekhet 2017a), mindfulness (Curl and Hampton 2023) and hope (Kulbaş and Özabacı 2022). Curl and Hampton also reported an increase in stress following their intervention.

#### 3.4.4 Spiritual Therapy

Pandya 2021 was the only study that focused on spiritual therapy. The intervention used spirituality as a supportive mechanism for family carers and included elements of mindfulness and self‐compassion. Pandya et al. conducted a RCT which met five (of seven) quality indicators according to the MMAT (Hong et al. 2018). The study focused on the effectiveness of the intervention and included care as usual as the control condition. They reported positive effects of the intervention on the stress of family carers.

#### 3.4.5 Expressive Writing

Whitney and Smith (2015) conducted a RCT focusing on the effectiveness of emotional disclosures through journal writing. The study met three (of seven) quality indicators according to the MMAT (Hong et al. 2018) and included a waitlist control group. Whitney and Smith reported that at post‐test participants in the intervention group reported higher levels of stress than the control group. This difference was no longer significant after controlling for baseline stress levels.

### 3.5 Secondary Outcomes

Twelve studies reported secondary outcomes (*n* = 7 mind–body, relaxation, mindfulness and acceptance interventions; *n* = 3 psychoeducation and support groups; *n* = 1 positive thinking and self‐compassion therapy, *n* = 1 spiritual therapy). See Table 2 for primary and secondary outcomes.

#### 3.5.1 Mind–Body, Relaxation, Mindfulness and Acceptance

Flujas‐Contreras et al. (2021) reported no significant change in children's behavioural problems at post‐test compared to baseline. Across both intervention arms (Be Mindful intervention with and without additional peer support), Flynn et al. (2020) reported significant decrease in participant‐child conflict, an increase in participant‐partner relationship, and participant‐partner agreement at post‐test compared to baseline. They reported no significant change for health‐related quality of life, participant‐child relationship, positive impact of the person with intellectual disability on the carer and family, parental sense of competence, parental efficacy and family functioning over time. Kuhlthau et al. (2020) found that at post‐test the intervention group experienced more significant improvements in social support compared to the control group. Lunsky et al. (2021) assessed mindful parenting and positive gains and reported improvements in both at post‐test compared to baseline. Padgett (2020) reported no significant change in mindful parenting between the intervention and control groups at post‐test. When comparing post‐test scores to baseline, Tilson (2022) found significant decrease in reported child difficulty as well as no significant changes in parent–child dysfunctional interactions and child's maladaptive, externalising and internalising behaviours. Finally, Zhou (2022) reported that when compared to the control group at post‐test, the intervention group experienced a more significant decrease in children's executive dysfunction, and an increase in mindful parenting. No significant changes in parental executive dysfunction were observed between intervention and control groups at post‐test.

#### 3.5.2 Psychoeducation and Support Groups

Bourke‐Taylor et al. (2022) reported significant differences between post‐test and baseline for children's quality of life and a subscale of Family Empowerment Scale. When compared with the control group, Hemdi and Daley (2017) found that the intervention group experienced significant decrease in parent–child dysfunctional interactions and improvement in children's emotional and behavioural functioning at post‐test. Kangavary et al. (2023) reported an increase in parenting sense of competence measure at post‐test compared to baseline.

#### 3.5.3 Positive Thinking and Self‐Compassion Therapy

Curl and Hampton (2023) found no significant change in parenting self‐efficacy at post‐test compared to baseline.

#### 3.5.4 Spiritual Therapy

Pandya (2021) reported a significant change at post‐test in child adjustment, parent efficacy, parenting resilience and maternal confidence for the intervention group.

### 3.6 Participants' Experiences and Factors Affecting the Implementation

Eighteen studies sought feedback from family carers about their experiences of taking part in interventions. All but one described findings. None of the studies sought opinions from people with intellectual and developmental disabilities. See Table S4 for a summary of family carer experiences and feedback reported in each study.

We narratively synthesised reported family carers' feedback. The identified broad topics were acceptability, facilitators, barriers and motivation to participate.

#### 3.6.1 Acceptability

Sixteen studies reported feedback related to the acceptability of the interventions. Interventions were generally reported as being acceptable to family carers. Participants appreciated the virtual format (*n* = 6 studies), would recommend the intervention to a friend (*n* = 4), found the intervention useful or valuable (*n* = 2), found time to engage with the intervention (*n* = 1) and enjoyed the topics and interactive activities (*n* = 1).

Where the intervention format allowed, participants reported that connecting with other caregivers as part of the intervention was helpful (Kuhlthau et al. 2020; Zimmerman 2013), and additional telephone calls from peer mentors were motivating and useful (Flynn et al. 2020).

#### 3.6.2 Facilitators of Implementation

Facilitators of implementation and participation were reported in 12 studies. Participants in eight studies reported that the virtual delivery of the interventions enabled them to access them, and that participation was easy, with some noting that while virtual delivery may be less personal, it is better than not being able to access support. The three studies which involved family carers in the design or delivery of their interventions reported co‐production was generally feasible and noted its importance. Tilson (2022) reported that their participants found shorter exercises, individualised sessions and privacy to discuss any topic during the sessions helpful. Other reported facilitators included: being able to access recordings of missed sessions (*n* = 3), having an intervention facilitator (*n* = 2), providing different relaxation strategies to choose from (*n* = 1), using applied activities rather than didactic teaching (*n* = 1), being able to complete the intervention at their own pace (*n* = 1) and incorporating real life examples (*n* = 1).

#### 3.6.3 Barriers to Implementation

Barriers to implementation and participation were reported in 10 studies. Some studies (*n* = 7) reported technology issues as a barrier to implementation of the intervention, with participants in one study (Luberto et al. 2021) reporting that the videoconferencing platform was distracting and unreliable. Participants in two studies reported difficulties with accessing technology. Curl and Hampton (2023) reported that there were difficulties with sharing resources online. Participants in five studies reported that it was difficult to find time to engage in the intervention or attend the intervention sessions, for example due to other commitments or childcare issues. Two studies using a group delivery mode reported that some participants found the content of the intervention not relevant to their experiences as family carers. This was usually due to the groups including parent carers of people with intellectual and developmental disabilities of different ages or different needs. Three studies reported that participants wanted more time to work on the intervention. Participants in other studies (*n* = 2) reported that inconsistent attendance and participation of other family carers in the intervention sessions was a barrier to their participation. Participants in Luberto et al. (2021) found completing homework stressful; they also reported that group video calls were uncomfortable or too personal and that remote delivery of the intervention did not encourage meaningful social connections. Bourke‐Taylor et al. (2022) included one face‐to‐face workshop as part of the intervention. Their participants reported difficulties in arranging childcare necessary to attend. Tilson (2022) used Theralytics to record data, and their participants reported difficulties with using the software.

#### 3.6.4 Motivation to Participate

Two studies (Clifford and Minnes 2013; Flynn et al. 2020) reported why participants were motivated to participate, and reported reasons were to reduce stress, improve wellbeing and self‐care. A less frequently reported motivation (*n* = 1) in these studies was to contribute to research.

## 4 Discussion

We found that 21 digital psychological wellbeing interventions for family carers of people with intellectual and developmental disabilities have been reported in the literature, and we categorised these broadly into five categories of interventions (mind–body, relaxation, mindfulness and acceptance; psychoeducation and support groups; positive thinking and self‐compassion; spiritual; and, expressive writing). Twenty‐three papers were identified within our systematic review evaluating the effectiveness of the 21 digital psychological wellbeing interventions. Eighteen papers sought feedback from family carers who had accessed the interventions, and the broad topics described were acceptability, facilitators, barriers and motivation to participate.

Evidence about the effectiveness and acceptability of the included digital wellbeing interventions for family carers of people with intellectual and developmental disabilities is generally promising, but still emerging. The strength of evidence within each of the five categories of intervention included in this review is variable, with no single intervention or category being identified as having strong evidence, and a lack of high‐quality RCTs (i.e., only Hemdi and Daley 2017 meeting six out of seven quality criteria) being employed to test the effectiveness and efficacy of digital wellbeing interventions for this population. The Hemdi and Daley RCT did not, however, report any data about participants' experiences of the intervention, indicating that further process evaluation work would be needed. It is, therefore, not possible at this stage to suggest that any of the included interventions would be suitably recommended as being useful in practice.

There is initially promising evidence about the effectiveness and acceptability of several interventions, including those at feasibility RCT stage (Flynn et al. 2020), and non‐RCT designs reporting positive changes to wellbeing post‐intervention (Bourke‐Taylor et al. 2022; Curl and Hampton 2023; Flujas‐Contreras et al. 2021; Kulbaş and Özabacı 2022; Lake et al. 2022; Luberto et al. 2021; Tilson 2022; Zimmerman 2013). Of these non‐RCT studies, the intervention tested in Bourke‐Taylor et al. (2022) was previously found to be effective in a face‐to‐face format, and so gathering additional effectiveness and acceptability data about the digital format would be a priority.

An overwhelming majority of included studies were published (or written, in the case of unpublished theses) after 2019, with some citing the COVID‐19 pandemic and the related restrictions as part of their rationale for digital delivery of the interventions. While it is very encouraging that researchers and practitioners adapted their practice to ensure therapeutic services remained accessible to family carers during the pandemic, this also suggests that digital delivery of many wellbeing interventions was mostly a response to the pandemic‐related restrictions, rather than the needs of the family carers. However, as difficulties attending face‐to‐face interventions are well documented in the literature (e.g., Lunsky et al. 2017) relating to family carers of children and adults with intellectual and developmental disabilities, it is important that increased digital access to psychological wellbeing interventions remains as a lasting legacy of the pandemic. While family carers in included studies reported some technical difficulties as barriers to implementation, the majority of feedback suggested that digital delivery of interventions was helpful and enabled participation where it would otherwise be difficult due to other, potentially more pervasive, barriers (e.g., transport, limited time, health problems). Further, the inclusion of an orientation session of the software used to deliver the sessions may offer a solution to most technical problems experienced within the reported interventions.

### 4.1 Recommendations for Implementing Wellbeing Interventions With Family Carers

Feedback from family carers who participated in the included studies suggests that interventions to improve their psychological wellbeing were generally acceptable; however, some challenges were present, and these were mostly related to the digital nature of the interventions rather than wider practical issues (e.g., arranging childcare). The barriers and facilitators reported within included papers were varied, indicating that family carers will have varying preferences and needs, further reinforcing the benefit of a wider variety of robustly evaluated, evidence‐based interventions available for family carers of children and adults with intellectual and developmental disabilities.

Based on feedback from the participants and authors of the included studies as well as reflections of our research team (which includes family carers), we came up with 12 recommendations for implementing digital wellbeing interventions with family carers (see Table 5). It is important to consider that the needs and preferences of family carers are diverse and depend on their situation; therefore, the flexibility to tailor adaptations and support for digital interventions to family carers' needs will likely enable more family carers to access them and benefit.

**TABLE 5:** Recommendations for implementing digital wellbeing interventions with family carers.

1.	Involve family carers in the development and delivery of the intervention.
2.	Incorporate real life examples, especially in relation to family carers of people of different ages and needs.
3.	Explore strategies that can facilitate interactions between family carers in a digital setting.
4.	Offer an initial orientation session to familiarise family carers with the software used and explore strategies to minimise on‐ and off‐screen distractions.
5.	Allow family carers to turn off their cameras during group sessions.
6.	Offer an option to stay longer after a synchronous facilitator‐led session to practice learnt strategies.
7.	Offer flexible delivery of interventions, including optional face‐to‐face sessions.
8.	Considering cultural adaptations.
9.	Incorporate reminders about upcoming sessions and homework.
10.	Offer access to recordings of synchronous sessions to family carers who missed them.
11.	Explore strategies to involve partners, co‐parents and other family members in the intervention, for example by developing written materials for them.
12.	Offer sessions at different times of the day.

When considering digitally delivered interventions, it is important to consider digital poverty as some families may not have access to electronic devices or the internet. One of the included studies had planned for this by providing family carers with the required resources for participation (Fenning et al. 2023). Ahmed and Raj (2023) suggested that intervention sessions for family carers could be embedded into appointments they attend with their loved ones with intellectual and developmental disabilities, for example, by giving them access to a tablet with intervention modules while in the waiting room. Offering this option to family carers could be a relatively low‐cost strategy to help with access to digital resources.

### 4.2 Strengths and Limitations of This Review

When interpreting the presented findings, it is important to also consider the limitations of the current review. Due to time constraints, it was not possible for two reviewers to independently extract data from the included papers. To ensure data accuracy, MA extracted data from all papers and AP checked the extracted information against the original sources. Initial screening was also not carried out by two reviewers due to the large number of records to screen.

To minimise the risk of missing potentially relevant research, we did not limit searches to papers available in English. This led to many non‐English papers being reviewed. While the research team has expertise in several languages apart from English (e.g., Polish, Greek), we used Google Translate to establish eligibility for some of the papers published in languages not known to the team. One paper published in Spanish was included in the systematic review (Flujas‐Contreras et al. 2021). Google Translate was also used to translate the paper for data extraction. As we were not able to validate this translation, some minor inaccuracies are possible. Finally, as with any systematic review, it is possible that some potentially relevant studies have been missed. However, the inclusion criteria and search strategy was designed to be broad enough to minimise this risk.

This review also had several strengths. The review protocol was published before searches started. We have followed PRISMA guidelines and best practice guides for systematic reviews in conducting the review and reporting the findings. Additionally, family carers of people with intellectual and developmental disabilities were members of the research team, ensuring involvement from people with lived experience.

### 4.3 Conclusions and Recommendations for Future Research

Digital interventions to improve wellbeing of family carers offer a promising alternative to face‐to‐face provisions, potentially allowing more family carers to access support. However, the quality of evidence is mixed. Whilst we included 10 RCTs in this review, given the generally limited number of high‐quality studies in this area, future research should continue to take robust, mixed methods (including RCT designs) approaches to evaluating interventions to build a strong evidence base. It is also crucial to consider the cultural context in which the intervention is being implemented. While studies included in the present review were conducted in various countries, the majority were from the USA. Before the implementation of any intervention, further adaptations will be needed to tailor the intervention to the cultural context in which it is being implemented to ensure it is relevant to family carers. Furthermore, co‐produced and co‐delivered interventions appeared to deliver positive effects, although only a small number of studies took this approach and no included interventions were solely delivered by family carers. This is a key area of development for future research investigating digital interventions to improve psychological wellbeing of family carers of children and adults with intellectual and developmental disabilities. Importantly, the varied nature of the interventions included in our review highlights that there is a need for family carers to have access to a variety of interventions to ensure that the varied needs of a diverse population of family carers are being met by these interventions.

## Author Contributions

All authors contributed to the study conception and design. Material preparation, data collection and analysis were performed by M.M.A., S.F. and A.P. The first draft of the manuscript was written by M.M.A. and S.F. J.G., R.P.H., E.F. and D.A. commented on drafts of the manuscript. All authors read and approved the final manuscript.

## Conflicts of Interest

S.F. and R.P.H. are the lead authors of one of the studies included in the review—Flynn et al. (2020). R.P.H. is also a co‐author of Lunsky et al. (2021). To manage the potential conflicts of interest, S.F. and R.P.H. were not involved in the study selection, data extraction, quality appraisal and data synthesis for those studies.

## Supporting information


**Data S1.** Supporting Information.

**Data S2.** Supporting Information.

**Data S3.** Supporting Information.

**Data S4.** Supporting Information.

**Data S5.** Supporting Information.

## Data Availability

The data that support the findings of this study are available from the corresponding author upon reasonable request.

## Endnotes

^1^
Whilst we recognise that neither person‐first nor identity‐first language are universally accepted, research suggests that identity‐first language is generally preferred by autistic people and person‐first language is generally preferred by people with intellectual disabilities. We will, therefore, be adopting this terminology within this paper.
