# Supplementary material for: Digital Psychological Wellbeing Interventions for Family Carers of Children and Adults With Intellectual and Developmental Disabilities: A Systematic Review
Source: J Appl Res Intellect Disabil. 2025 Jul 11;38(4):e70081. doi: 10.1111/jar.70081 (PMC12247015; doi:10.1111/jar.70081)
Supplement: Supplementary file 2 — Data S2. Supporting Information. [file JAR-38-e70081-s002.docx]

Supplementary document 2

Search strings.

| **List 1** | **List 2** | **List 3** | **LIST 4** |
| --- | --- | --- | --- |
| **MEDLINE (including Pubmed) via OVID** | | | |
| Intellectual* Disab* OR Intellectual* Impairment* OR Intellectual* Deficien* OR Intellectual* Handicap* OR Intellectual* Subnormal* OR Intellectual* Retard* OR Intellectual* Difficult* OR Learning Disab* OR Learning Impairment* OR Learning Deficien* OR Learning Handicap* OR Learning Subnormal* OR Learning Retard* OR Learning Difficult* OR Mental* Disab* OR Mental* Impairment* OR Mental* Deficien* OR Mental* Handicap* OR Mental* Subnormal* OR Mental* Retard* OR Mental* Difficult* OR Developmental Disab* OR Developmental Impairment* OR Developmental Deficien* OR Developmental Handicap* OR Developmental Subnormal* OR Developmental Retard* OR Developmental Difficult* OR Down Syndrome OR Asperger OR Autis* OR Autism Spectrum Disorder OR ASD OR Pervasive Developmental Disorder OR PDD OR Smith-Magenis OR Rett* OR Lesch-Nyhan OR Prader-Willi OR Angelman OR fragile X OR Cri-du-chat OR Cornelia de Lange OR de Lange OR Rubinstein-Taybi OR velocardiofacial OR DiGeorge OR Down* OR Fetal alcohol OR Overgrowth syndrome OR Neurodevelopmental | online intervention* OR online treatment* OR online therap* OR program OR digital intervention* OR digital treatment* OR digital therap* OR digital program mobile intervention* OR mobile treatment* OR mobile therap* OR mobile program OR virtual OR smartphone intervention* OR smartphone treatment* OR smartphone therap* OR web-based intervention* OR web-based treatment* OR web based therap* OR internet intervention* OR internet treatment* OR internet therap* OR internet-based OR internet based OR cyber* OR cyber intervention* OR cyber treatment* OR cyber therap* OR mhealth OR ehealth OR mtherap* OR etherap* OR m-health OR e-health OR m therap* OR e-therap* OR Tele* OR telehealth OR telemedicine OR digital health OR mobile app OR app OR app-based | parent* OR carer* OR care* OR family carer* OR family member* OR mother* OR father* OR guardian* OR sibling* OR adopt* OR step-parent* OR step parent* OR step-father* OR step father* OR step-mother* OR step mother* | wellbeing OR well-being OR mental health OR quality of life OR QoL OR happiness OR life satisfaction OR stress OR burnout OR burn-out OR anxiety OR anxious OR depress* OR mental* ill* OR posttraumatic stress OR post-traumatic stress OR resilien* OR parent* coping OR care* copying OR parent* satisfaction OR care* satisfaction OR psychologic* flexib* OR loneliness OR burden OR distress adj2 psychologic* OR distress adj2 mental |
| **EMBASE via OVID** | | | |
| Intellectual* Disab* OR Intellectual* Impairment* OR Intellectual* Deficien* OR Intellectual* Handicap* OR Intellectual* Subnormal* OR Intellectual* Retard* OR Intellectual* Difficult* OR Learning Disab* OR Learning Impairment* OR Learning Deficien* OR Learning Handicap* OR Learning Subnormal* OR Learning Retard* OR Learning Difficult* OR Mental* Disab* OR Mental* Impairment* OR Mental* Deficien* OR Mental* Handicap* OR Mental* Subnormal* OR Mental* Retard* OR Mental* Difficult* OR Developmental Disab* OR Developmental Impairment* OR Developmental Deficien* OR Developmental Handicap* OR Developmental Subnormal* OR Developmental Retard* OR Developmental Difficult* OR Down Syndrome OR Asperger OR Autis* OR Autism Spectrum Disorder OR ASD OR Pervasive Developmental Disorder OR PDD OR Smith-Magenis OR Rett* OR Lesch-Nyhan OR Prader-Willi OR Angelman OR fragile X OR Cri-du-chat OR Cornelia de Lange OR de Lange OR Rubinstein-Taybi OR velocardiofacial OR DiGeorge OR Down* OR Fetal alcohol OR Overgrowth syndrome OR Neurodevelopmental | online intervention* OR online treatment* OR online therap* OR program OR digital intervention* OR digital treatment* OR digital therap* OR digital program mobile intervention* OR mobile treatment* OR mobile therap* OR mobile program OR virtual OR smartphone intervention* OR smartphone treatment* OR smartphone therap* OR web-based intervention* OR web-based treatment* OR web based therap* OR internet intervention* OR internet treatment* OR internet therap* OR internet-based OR internet based OR cyber* OR cyber intervention* OR cyber treatment* OR cyber therap* OR mhealth OR ehealth OR mtherap* OR etherap* OR m-health OR e-health OR m therap* OR e-therap* OR Tele* OR telehealth OR telemedicine OR digital health OR mobile app OR app OR app-based | parent* OR carer* OR care* OR family carer* OR family member* OR mother* OR father* OR guardian* OR sibling* OR adopt* OR step-parent* OR step parent* OR step-father* OR step father* OR step-mother* OR step mother* | wellbeing OR well-being OR mental health OR quality of life OR QoL OR happiness OR life satisfaction OR stress OR burnout OR burn-out OR anxiety OR anxious OR depress* OR mental* ill* OR posttraumatic stress OR post-traumatic stress OR resilien* OR parent* coping OR care* copying OR parent* satisfaction OR care* satisfaction OR psychologic* flexib* OR loneliness OR burden OR distress adj2 psychologic* OR distress adj2 mental |
| **PsycINFO via OVID** | | | |
| Intellectual* Disab* OR Intellectual* Impairment* OR Intellectual* Deficien* OR Intellectual* Handicap* OR Intellectual* Subnormal* OR Intellectual* Retard* OR Intellectual* Difficult* OR Learning Disab* OR Learning Impairment* OR Learning Deficien* OR Learning Handicap* OR Learning Subnormal* OR Learning Retard* OR Learning Difficult* OR Mental* Disab* OR Mental* Impairment* OR Mental* Deficien* OR Mental* Handicap* OR Mental* Subnormal* OR Mental* Retard* OR Mental* Difficult* OR Developmental Disab* OR Developmental Impairment* OR Developmental Deficien* OR Developmental Handicap* OR Developmental Subnormal* OR Developmental Retard* OR Developmental Difficult* OR Down Syndrome OR Asperger OR Autis* OR Autism Spectrum Disorder OR ASD OR Pervasive Developmental Disorder OR PDD OR Smith-Magenis OR Rett* OR Lesch-Nyhan OR Prader-Willi OR Angelman OR fragile X OR Cri-du-chat OR Cornelia de Lange OR de Lange OR Rubinstein-Taybi OR velocardiofacial OR DiGeorge OR Down* OR Fetal alcohol OR Overgrowth syndrome OR Neurodevelopmental | online intervention* OR online treatment* OR online therap* OR program OR digital intervention* OR digital treatment* OR digital therap* OR digital program mobile intervention* OR mobile treatment* OR mobile therap* OR mobile program OR virtual OR smartphone intervention* OR smartphone treatment* OR smartphone therap* OR web-based intervention* OR web-based treatment* OR web based therap* OR internet intervention* OR internet treatment* OR internet therap* OR internet-based OR internet based OR cyber* OR cyber intervention* OR cyber treatment* OR cyber therap* OR mhealth OR ehealth OR mtherap* OR etherap* OR m-health OR e-health OR m therap* OR e-therap* OR Tele* OR telehealth OR telemedicine OR digital health OR mobile app OR app OR app-based | parent* OR carer* OR care* OR family carer* OR family member* OR mother* OR father* OR guardian* OR sibling* OR adopt* OR step-parent* OR step parent* OR step-father* OR step father* OR step-mother* OR step mother* | wellbeing OR well-being OR mental health OR quality of life OR QoL OR happiness OR life satisfaction OR stress OR burnout OR burn-out OR anxiety OR anxious OR depress* OR mental* ill* OR posttraumatic stress OR post-traumatic stress OR resilien* OR parent* coping OR care* copying OR parent* satisfaction OR care* satisfaction OR psychologic* flexib* OR loneliness OR burden OR distress adj2 psychologic* OR distress adj2 mental |
| **Scopus** | | | |
| "Intellectual* Disab*” OR “Intellectual* Impairment*” OR “Intellectual* Deficien*” OR “Intellectual* Handicap*” OR “Intellectual* Subnormal*” OR “Intellectual* Retard*” OR “Intellectual* Difficult*" OR "Learning Disab*” OR "Learning Impairment*” OR "Learning Deficien*” OR "Learning Handicap*” OR "Learning Subnormal*” OR "Learning Retard*” OR "Learning Difficult*" OR "Mental* Disab*” OR "Mental* Impairment*” OR "Mental* Deficien*” OR "Mental* Handicap*” OR "Mental* Subnormal*” OR "Mental* Retard*” OR "Mental* Difficult*" OR "Developmental Disab*” OR "Developmental Impairment*” OR "Developmental Deficien*” OR "Developmental Handicap*” OR "Developmental Subnormal*” OR "Developmental Retard*” OR "Developmental Difficult*" OR "Down Syndrome" OR Asperger OR Autis* OR "Autism Spectrum Disorder" OR ASD OR "Pervasive Developmental Disorder" OR PDD OR "Smith-Magenis" OR Rett* OR "Lesch-Nyhan" OR "Prader-Willi" OR Angelman OR "fragile X" OR "Cri-du-chat" OR "Cornelia de Lange" OR "de Lange" OR "Rubinstein-Taybi" OR velocardiofacial OR DiGeorge OR Down* OR "Fetal alcohol" OR "Overgrowth syndrome" OR Neurodevelopmental | “online intervention*” OR “online treatment*” OR “online therap*” OR program OR “digital intervention*” OR “digital treatment*” OR “digital therap*” OR “digital program mobile intervention*” OR “mobile treatment*” OR “mobile therap*” OR “mobile program” OR virtual OR “smartphone intervention*” OR “smartphone treatment*” OR “smartphone therap*” OR “web-based intervention*” OR “web-based treatment*” OR “web based therap*” OR“internet intervention*” OR “internet treatment*” OR “internet therap*” OR “internet-based” OR “internet based” OR cyber* OR “cyber intervention*” OR “cyber treatment*” OR “cyber therap*” OR mhealth OR ehealth OR mtherap* OR etherap* OR “m-health” OR “e-health” OR “m therap*” OR “e-therap*” OR Tele* OR telehealth OR telemedicine OR “digital health” OR “mobile app” OR app OR “app-based” | parent* OR carer* OR care* OR “family carer*” OR ”family member*” OR mother* OR father* OR guardian* OR sibling* OR adopt* OR “step-parent*” OR “step parent*” OR “step-father*” OR “step father*” OR “step-mother*” OR “step mother*” | wellbeing OR “well-being” OR “mental health” OR “quality of life” OR QoL OR happiness OR “life satisfaction” OR stress OR burnout OR “burn-out” OR anxiety OR anxious OR depress* OR “mental* ill*” OR “posttraumatic stress” OR “post-traumatic stress” OR resilien* OR “parent* coping” OR “care* copying” OR “parent* satisfaction” OR “care* satisfaction” OR “psychologic* flexib*” OR loneliness OR burden OR “distress W/2 psychologic*” OR “distress W/2 mental” |
| **Web of Science (all databases)** | | | |
| "Intellectual* Disab*” OR “Intellectual* Impairment*” OR “Intellectual* Deficien*” OR “Intellectual* Handicap*” OR “Intellectual* Subnormal*” OR “Intellectual* Retard*” OR “Intellectual* Difficult*" OR "Learning Disab*” OR "Learning Impairment*” OR "Learning Deficien*” OR "Learning Handicap*” OR "Learning Subnormal*” OR "Learning Retard*” OR "Learning Difficult*" OR "Mental* Disab*” OR "Mental* Impairment*” OR "Mental* Deficien*” OR "Mental* Handicap*” OR "Mental* Subnormal*” OR "Mental* Retard*” OR "Mental* Difficult*" OR "Developmental Disab*” OR "Developmental Impairment*” OR "Developmental Deficien*” OR "Developmental Handicap*” OR "Developmental Subnormal*” OR "Developmental Retard*” OR "Developmental Difficult*" OR "Down Syndrome" OR Asperger OR Autis* OR "Autism Spectrum Disorder" OR ASD OR "Pervasive Developmental Disorder" OR PDD OR "Smith-Magenis" OR Rett* OR "Lesch-Nyhan" OR "Prader-Willi" OR Angelman OR "fragile X" OR "Cri-du-chat" OR "Cornelia de Lange" OR "de Lange" OR "Rubinstein-Taybi" OR velocardiofacial OR DiGeorge OR Down* OR "Fetal alcohol" OR "Overgrowth syndrome" OR Neurodevelopmental | “online intervention*” OR “online treatment*” OR “online therap*” OR program OR “digital intervention*” OR “digital treatment*” OR “digital therap*” OR “digital program mobile intervention*” OR “mobile treatment*” OR “mobile therap*” OR “mobile program” OR virtual OR “smartphone intervention*” OR “smartphone treatment*” OR “smartphone therap*” OR “web-based intervention*” OR “web-based treatment*” OR “web based therap*” OR“internet intervention*” OR “internet treatment*” OR “internet therap*” OR “internet-based” OR “internet based” OR cyber* OR “cyber intervention*” OR “cyber treatment*” OR “cyber therap*” OR mhealth OR ehealth OR mtherap* OR etherap* OR “m-health” OR “e-health” OR “m therap*” OR “e-therap*” OR Tele* OR telehealth OR telemedicine OR “digital health” OR “mobile app” OR app OR “app-based” | parent* OR carer* OR care* OR “family carer*” OR ”family member*” OR mother* OR father* OR guardian* OR sibling* OR adopt* OR “step-parent*” OR “step parent*” OR “step-father*” OR “step father*” OR “step-mother*” OR “step mother*” | wellbeing OR “well-being” OR “mental health” OR “quality of life” OR QoL OR happiness OR “life satisfaction” OR stress OR burnout OR “burn-out” OR anxiety OR anxious OR depress* OR “mental* ill*” OR “posttraumatic stress” OR “post-traumatic stress” OR resilien* OR “parent* coping” OR “care* copying” OR “parent* satisfaction” OR “care* satisfaction” OR “psychologic* flexib*” OR loneliness OR burden OR “distress NEAR2 psychologic*” OR “distress NEAR2 mental” |
| **CINAHL** | | | |
| "Intellectual* Disab*” OR “Intellectual* Impairment*” OR “Intellectual* Deficien*” OR “Intellectual* Handicap*” OR “Intellectual* Subnormal*” OR “Intellectual* Retard*” OR “Intellectual* Difficult*" OR "Learning Disab*” OR "Learning Impairment*” OR "Learning Deficien*” OR "Learning Handicap*” OR "Learning Subnormal*” OR "Learning Retard*” OR "Learning Difficult*" OR "Mental* Disab*” OR "Mental* Impairment*” OR "Mental* Deficien*” OR "Mental* Handicap*” OR "Mental* Subnormal*” OR "Mental* Retard*” OR "Mental* Difficult*" OR "Developmental Disab*” OR "Developmental Impairment*” OR "Developmental Deficien*” OR "Developmental Handicap*” OR "Developmental Subnormal*” OR "Developmental Retard*” OR "Developmental Difficult*" OR "Down Syndrome" OR Asperger OR Autis* OR "Autism Spectrum Disorder" OR ASD OR "Pervasive Developmental Disorder" OR PDD OR "Smith-Magenis" OR Rett* OR "Lesch-Nyhan" OR "Prader-Willi" OR Angelman OR "fragile X" OR "Cri-du-chat" OR "Cornelia de Lange" OR "de Lange" OR "Rubinstein-Taybi" OR velocardiofacial OR DiGeorge OR Down* OR "Fetal alcohol" OR "Overgrowth syndrome" OR Neurodevelopmental | “online intervention*” OR “online treatment*” OR “online therap*” OR program OR “digital intervention*” OR “digital treatment*” OR “digital therap*” OR “digital program mobile intervention*” OR “mobile treatment*” OR “mobile therap*” OR “mobile program” OR virtual OR “smartphone intervention*” OR “smartphone treatment*” OR “smartphone therap*” OR “web-based intervention*” OR “web-based treatment*” OR “web based therap*” OR“internet intervention*” OR “internet treatment*” OR “internet therap*” OR “internet-based” OR “internet based” OR cyber* OR “cyber intervention*” OR “cyber treatment*” OR “cyber therap*” OR mhealth OR ehealth OR mtherap* OR etherap* OR “m-health” OR “e-health” OR “m therap*” OR “e-therap*” OR Tele* OR telehealth OR telemedicine OR “digital health” OR “mobile app” OR app OR “app-based” | parent* OR carer* OR care* OR “family carer*” OR ”family member*” OR mother* OR father* OR guardian* OR sibling* OR adopt* OR “step-parent*” OR “step parent*” OR “step-father*” OR “step father*” OR “step-mother*” OR “step mother*” | wellbeing OR “well-being” OR “mental health” OR “quality of life” OR QoL OR happiness OR “life satisfaction” OR stress OR burnout OR “burn-out” OR anxiety OR anxious OR depress* OR “mental* ill*” OR “posttraumatic stress” OR “post-traumatic stress” OR resilien* OR “parent* coping” OR “care* copying” OR “parent* satisfaction” OR “care* satisfaction” OR “psychologic* flexib*” OR loneliness OR burden OR “distress N2 psychologic*” OR “distress N2 mental” |
| **Applied Social Sciences Index and Abstracts (ASSIA)** | | | |
| "Intellectual Disability” OR "Intellectually Disabled” OR “Intellectual Impairment” OR “Intellectual Impairments” OR “Intellectual Deficient” OR “Intellectual Deficiency” OR “Intellectually Deficient” OR “Intellectual Handicap” OR “Intellectually Handicaped” OR “Intellectual Subnormal” OR “Intellectually Subnormal” OR “Intellectual Subnormality” OR “Intellectual Retard” OR “Intellectually Retarded” OR “Intellectual Difficulty" OR “Intellectually Difficult" OR "Learning Disability” OR "Learning Disabled” OR "Learning Impairment” OR "Learning Impairments” OR "Learning Deficient” OR "Learning Deficiency” OR "Learning Handicap” OR "Learning Handicapped” OR "Learning Subnormal” OR "Learning Subnormality” OR "Learning Retard” OR "Learning Retardation” OR "Learning Difficulty" OR "Learning Difficult" OR "Learning Difficulties" OR "Mental Disability” OR "Mentally Disabled” OR "Mental Impairment” OR "Mental Impairments” OR "Mental Deficiency” OR "Mentally Deficient” OR "Mental Handicap” OR "Mentally Handicapped” OR "Mentally Subnormal” OR "Mental Subnormality” OR "Mental Retardation” OR "Mentally Retarded” OR "Mental Difficulty" OR "Mentally Difficult" OR "Developmental Disability” OR "Developmentally Disabled” OR "Developmental Disabilities” OR "Developmental Impairment” OR "Developmental Impairments” OR "Developmental Deficiency” OR "Developmental Deficiencies” OR "Developmental Handicap” OR "Developmental Handicaps” OR "Developmental Subnormalities” OR "Developmental Subnormality” OR "Developmental Retard” OR "Developmental Retardation” OR "Developmental Difficultity" OR "Developmental Difficulties" OR "Down Syndrome" OR Asperger OR Autis* OR "Autism Spectrum Disorder" OR ASD OR "Pervasive Developmental Disorder" OR PDD OR "Smith-Magenis" OR Rett* OR "Lesch-Nyhan" OR "Prader-Willi" OR Angelman OR "fragile X" OR "Cri-du-chat" OR "Cornelia de Lange" OR "de Lange" OR "Rubinstein-Taybi" OR velocardiofacial OR DiGeorge OR Down* OR "Fetal alcohol" OR "Overgrowth syndrome" OR Neurodevelopmental | “online intervention” OR “online interventions” OR “online treatment” OR “online treatments” OR “online therapy” OR “online therapist” OR program OR “digital intervention” OR “digital interventions” OR “digital treatment” OR “digital treatments” OR “digital therapy” OR “digital therapist” OR “digital program mobile intervention” OR “digital program mobile interventions” OR “mobile treatment” OR “mobile treatments” OR “mobile therapy” OR “mobile therapist” OR “mobile program” OR virtual OR “smartphone intervention” OR “smartphone interventions” OR “smartphone treatment” OR “smartphone treatments” OR “smartphone therapy” OR “smartphone therapist” OR “web-based intervention” OR “web-based interventions” OR “web-based treatment” OR “web-based treatments” OR “web based therapy” OR “web based therapist*” OR “internet intervention” OR “internet interventions” OR “internet treatment” OR “internet treatments” OR “internet therapy” OR “internet therapist” OR “internet-based” OR “internet based” OR cyber* OR “cyber intervention” OR “cyber interventions” OR “cyber treatment” OR “cyber treatments” OR “cyber therapy” OR “cyber therapist” OR mhealth OR ehealth OR mtherap* OR etherap* OR “m-health” OR “e-health” OR “m therapist” OR “m therapy” OR “e-therapy” OR “e-therapist” OR Tele* OR telehealth OR telemedicine OR “digital health” OR “mobile app” OR app OR “app-based” | parent* OR carer* OR care* OR “family carer” OR “family carers” OR ”family member” OR ”family members” OR mother* OR father* OR guardian* OR sibling* OR adopt* OR “step-parent” OR “step-parents” OR “step parent” OR “step parents” OR “step-father” OR “step-fathers”OR “step father” OR “step fathers” OR “step-mother” OR “step-mothers” OR “step mother” OR “step mothers” | wellbeing OR “well-being” OR “mental health” OR “quality of life” OR QoL OR happiness OR “life satisfaction” OR stress OR burnout OR “burn-out” OR anxiety OR anxious OR depress* OR “mental illness” OR “mentally ill” OR “posttraumatic stress” OR “post-traumatic stress” OR resilien* OR “parent coping” OR “parental coping” OR “carer copying” OR “carers copying” OR “parent satisfaction” OR “parental satisfaction” OR “carer satisfaction” OR “carers satisfaction” “psychologic flexibility” OR “psychological flexibility” OR “psychologically flexible” OR loneliness OR burden OR “psychologic distress” OR “psychological distress” OR “mental distress” |
