# Supplementary material for: Digital Psychological Wellbeing Interventions for Family Carers of Children and Adults With Intellectual and Developmental Disabilities: A Systematic Review
Source: J Appl Res Intellect Disabil. 2025 Jul 11;38(4):e70081. doi: 10.1111/jar.70081 (PMC12247015; doi:10.1111/jar.70081)
Supplement: Supplementary file 3 — Data S3. Supporting Information. [file JAR-38-e70081-s005.docx]

Supplementary document 3

List of papers excluded after full text review.

Abdoli, F., Rafiean, S., & Haji-Adineh, S. (2019). The effect of cognitive-behavioral stress management training on psychological health and stress among parents of mentally disabled children. International Journal of Body, Mind and Culture, 6(3), 152-159. https://doi.org/doi:10.22122/ijbmc.v6i3.179

Akdogan, R. (2016). A Holistic Approach to Cope with Depression and Hopelessness for Parents of Special Needs Children. INTERNATIONAL JOURNAL OF EARLY CHILDHOOD SPECIAL EDUCATION, 8(2), 134-150. https://doi.org/doi:10.20489/intjecse.284594

Alaedein, J. M., & Al-Sharaa, F. K. (2020). The effect of group counseling in reducing parental stress and depression in jordanian mothers of children with autism. International Journal of Education and Practice, 8(3), 518-535. https://doi.org/doi:10.18488/journal.61.2020.83.518.535

Al-Khalaf, A., Dempsey, I., & Dally, K. (2014). The effect of an education program for mothers of children with Autism Spectrum Disorder in Jordan. INTERNATIONAL JOURNAL FOR THE ADVANCEMENT OF COUNSELLING, 36(2), 175-187. https://doi.org/doi:https://dx.doi.org/10.1007/s10447-013-9199-3

Al-Masa'deh, M. M., Younis, N. A., Al-Zoud, N. S., Homidi, M. A., & Rahahleh, Z. J. (2022). THE EFFECTIVENESS OF COUNSELING PROGRAMS IN ENHANCING QUALITY OF LIFE FOR FAMILIES OF CHILDREN WITH AUTISM SPECTRUM DISORDER IN JORDAN. Humanities and Social Sciences Letters, 10(1), 75-87. https://doi.org/doi:10.18488/73.v10i1.2944

Al-quran, N. M., Salameh, M. S. B., & Alradi, M. N. M. (2023). The Effect of a Family Counseling Program in Reducing Stress in Mothers of Children with Autism Spectrum Disorder [Article]. Journal for ReAttach Therapy and Developmental Diversities, 6(8), 350-364. https://www.scopus.com/inward/record.uri?eid=2-s2.0-85165967719&partnerID=40&md5=807a6d7d888cd4146d79b1285cc8705a

Anclair, M., Hjärthag, F., & Hiltunen, A. J. (2017). Cognitive behavioural therapy and mindfulness for health-related quality of life: Comparing treatments for parents of children with chronic conditions - A pilot feasibility study. Clinical Practice and Epidemiology in Mental Health, 13, 1-9. https://doi.org/doi:10.2174/1745017901713010001

Andrews, M. L., Garcia, Y. A., Catagnus, R. M., & Gould, E. R. (2022). Effects of Acceptance and Commitment Training Plus Behavior Parent Training on Parental Implementation of Autism Treatment. The Psychological record, 72(4), 601-617. https://doi.org/doi:https://dx.doi.org/10.1007/s40732-021-00496-5

Appleton, P., Barrasin, E., Hepworth, A., Appleton, D., Lesley, R., & Melluish, S. (2020). Effects of a mindfulness-based stress reduction group programme on family carers. Mental Health Practice, 23(1), 17-22. https://doi.org/doi:10.7748/mhp.2019.e1362

Ardic, A., & Cavkaytar, A. (2019). The Effect of the Psychoeducational Group Family Education Program for Families of Children Diagnosed with Autism Spectrum Disorder on Parents: A Pilot Study. INTERNATIONAL JOURNAL OF EARLY CHILDHOOD SPECIAL EDUCATION, 11(1), 1-17. https://doi.org/doi:10.20489/intjecse.581495

Arthur, K. C., Sanchez, A. M., Montero, A. T., Delgado, P., Ramos, O. R., Orlich, F., & Desai, A. D. (2024). Identifying adaptations for a mindfulness program for Spanish-speaking mothers of children with chronic conditions or disabilities. HEALTH EDUCATION RESEARCH. https://doi.org/10.1093/her/cyad043

Arzhangi, S., Hosseini, M., Hosseinzadeh, S., & Tafreshi, M. Z. (2019). The Effects of Time Use Training on the Level of Stress among the Mothers of Female Children with Intellectual Disabilities. NURSING AND MIDWIFERY STUDIES, 8(3), 126-131. https://doi.org/doi:10.4103/nms.nms_27_18

Ashori, M., Norouzi, G., & Jalil-Abkenar, S. S. (2019). The effect of positive parenting program on mental health in mothers of children with intellectual disability. Journal of intellectual disabilities : JOID, 23(3), 385-396. https://doi.org/doi:https://dx.doi.org/10.1177/1744629518824899

Askari, S., Hassanpour, T., Shirvani, Z., Eslamiyan, S., & Gholamnezhad, M. (2022). Health Education and quality of life of mothers with mentally retarded daughters. NeuroQuantology, 20(16), 4900-4904. https://doi.org/doi:https://dx.doi.org/10.48047/NQ.2022.20.16.NQ880497

Bazzano, A., Wolfe, C., Zylowska, L., Wang, S., Schuster, E., Barrett, C., & Lehrer, D. (2015). Mindfulness based stress reduction (MBSR) for parents and caregivers of individuals with developmental disabilities: A community-based approach. JOURNAL OF CHILD AND FAMILY STUDIES, 24(2), 298-308. https://doi.org/doi:https://dx.doi.org/10.1007/s10826-013-9836-9

Bedard, K. E., Griffith, A. K., Ulm, D., Strittman, M., Krukowski, K., Eaton, A., Rone, A., & Cardon, T. (2023). Evaluating the Impact of PWS Smart-Start: A Behavior Analytic Caregiver Training Program for Prader-Willi Syndrome [Article]. Journal of Positive Behavior Interventions. https://doi.org/10.1177/10983007231200535

Behar-Seiloe, S. (2020). Grief and anxiety in parents of children with autism. Dissertation Abstracts International Section A: Humanities and Social Sciences, 81(2), No-Specified. https://doi.org/doi:

Bharat, R., Uzaina, U., Yadav, T., Niranjan, S., Qazi, S., & Gopal, N. (2023). App to Target Parental Understanding and Expectation: A Game Changer in Neurodevelopmental Conditions. Developmental Medicine and Child Neurology, 65, 111. https://doi.org/doi:https://dx.doi.org/10.1111/dmcn.15477

Bilgin, S., & Gozum, S. (2009). Reducing burnout in mothers with an intellectually disabled child: an education programme. Journal of advanced nursing, 65(12), 2552-2561. https://doi.org/doi:https://dx.doi.org/10.1111/j.1365-2648.2009.05163.x

Bitsika, V., & Sharpley, C. (1999). An explanatory examination of the effects of support groups on the well-being of parents of children with autism: I: General counselling. Journal of Applied Health Behaviour, 1(2), 16-22. https://doi.org/doi:

Bitsika, V., & Sharpley, C. (2000). Development and testing of the effects of support groups on the well-being of parents of children with autism-II: Specific stress management techniques. Journal of Applied Health Behaviour, 2(1), 8-15. https://doi.org/doi:

Boezeman, E. J., Nieuwenhuijsen, K., & Sluiter, J. K. (2018). An intervention that reduces stress in people who combine work with informal care: randomized controlled trial results. EUROPEAN JOURNAL OF PUBLIC HEALTH, 28(3), 485-489. https://doi.org/doi:10.1093/eurpub/cky052

Bourke-Taylor, H. M., & Jane, F. M. (2018). Mothers' Experiences of a Women's Health and Empowerment Program for Mothers of a Child with a Disability. JOURNAL OF AUTISM AND DEVELOPMENTAL DISORDERS, 48(6), 2174-2186. https://doi.org/doi:10.1007/s10803-018-3486-0

Bourke-Taylor, H. M., Jane, F., & Peat, J. (2019). Healthy Mothers Healthy Families Workshop Intervention: A Preliminary Investigation of Healthy Lifestyle Changes for Mothers of a Child with a Disability. JOURNAL OF AUTISM AND DEVELOPMENTAL DISORDERS, 49(3), 935-949. https://doi.org/doi:10.1007/s10803-018-3789-1

Bourke-Taylor, H., Leo, M., & Tirlea, L. (2023). Health, Wellbeing and Empowerment E-workshops for Mothers of Children with Disabilities: A non-randomised comparison study.

Bourke-Taylor, H., Leo, M., Harris, V., & Tirlea, L. (2023). Feasibility of Health Promoting Activity Coaching for Mothers of Children With Disabilities: Pilot Nonrandomized Controlled Trial

Boursier, V., Gioia, F., Coppola, F., & Schimmenti, A. (2022). eHealth content-sharing and emotional support among Italian parents of children with Down Syndrome: A qualitative report. JOURNAL OF INTELLECTUAL & DEVELOPMENTAL DISABILITY, 47(3), 240-251. https://doi.org/doi:10.3109/13668250.2021.1964153

Bravo-Benítez, J., Cruz-Quintana, F., Navarro, E., & Pérez-Marfil, M. N. (2024). Grief Intervention Program for Caregivers to Individuals with Autism Spectrum Disorder (ASD): A Randomized Preliminary Trial [Article]. Journal of Child and Family Studies. https://doi.org/10.1007/s10826-023-02750-6

Brookman-Frazee, L., Chlebowski, C., Villodas, M., Roesch, S., & Martinez, K. (2021). Training Community Therapists to Deliver an Individualized Mental Health Intervention for Autism Spectrum Disorder: Changes in Caregiver Outcomes and Mediating Role on Child Outcomes. JOURNAL OF THE AMERICAN ACADEMY OF CHILD AND ADOLESCENT PSYCHIATRY, 60(3), 355-366. https://doi.org/doi:10.1016/j.jaac.2020.07.896

Brouzos, A., Vassilopoulos, S. P., & Tassi, C. (2017). A Psychoeducational Group Intervention for Siblings of Children With Autism Spectrum Disorder. JOURNAL FOR SPECIALISTS IN GROUP WORK, 42(4), 274-298. https://doi.org/doi:10.1080/01933922.2017.1350230

Burke, M. M., Chan, N., & Neece, C. L. (2017). Parent Perspectives of Applying Mindfulness-Based Stress Reduction Strategies to Special Education. INTELLECTUAL AND DEVELOPMENTAL DISABILITIES, 55(3), 167-180. https://doi.org/doi:10.1352/1934-9556-55.3.167

Burkhart, K., Lipman, C., Agarwal, N., Tangen, R., & Bothe, D. (2024). Fetal alcohol spectrum disorder: an exploratory telehealth pilot group intervention for caregivers. CHILDRENS HEALTH CARE. https://doi.org/10.1080/02739615.2023.2300648

Byrne, G., Vickers, L., Ni Longphuirt, E., & Cunningham, R. (2022). Evaluation of Telehealth Delivery of Group Parent-Led Cognitive-Behavioral Therapy During COVID-19: A Pilot Study. Focus on Autism and Other Developmental Disabilities, 37(4), 251-256. https://doi.org/doi:https://dx.doi.org/10.1177/10883576221121280

Caldwell, J., & Heller, T. (2007). Longitudinal outcomes of a consumer-directed program supporting adults with developmental disabilities and their families. INTELLECTUAL AND DEVELOPMENTAL DISABILITIES, 45(3), 161-173. https://doi.org/doi:

Canon, L. F., Gould, E. R., Sandoz, E. K., Moran, O., & Grimaldi, M. A. (2023). Cultural adaptation of ACT to support caregivers of autistic Latino children: A pilot study. JOURNAL OF CONTEXTUAL BEHAVIORAL SCIENCE, 28, 1-9. https://doi.org/doi:https://dx.doi.org/10.1016/j.jcbs.2023.03.003

Cappe, E., Downes, N., Albert-Benaroya, S., Ech-Chouikh, J. A., De Gaulmyn, A., Luperto, L., Caron, V., Roussel, E., Taton, R., & Sankey, C. (2021). Preliminary Results of the Effects of a Psychoeducational Program on Stress and Quality of Life Among French Parents of an Child With Autism. Focus on Autism and Other Developmental Disabilities, 36(3), 176-186. https://doi.org/doi:https://dx.doi.org/10.1177/1088357620986946

Carlier, S., Van der Paelt, S., Ongenae, F., De Backere, F., & De Turck, F. (2020). Empowering Children with ASD and Their Parents: Design of a Serious Game for Anxiety and Stress Reduction. Sensors (Basel, Switzerland), 20(4). https://doi.org/doi:https://dx.doi.org/10.3390/s20040966

Chaudhry, N., Sattar, R., Kiran, T., Wan, M. W., Husain, M., Hidayatullah, S., Ali, B., Shafique, N., Suhag, Z., Saeed, Q., Maqbool, S., & Husain, N. (2023). Supporting Depressed Mothers of Young Children with Intellectual Disability: Feasibility of an Integrated Parenting Intervention in a Low-Income Setting [Article]. Children, 10(6). https://doi.org/10.3390/children10060913

Chiang, H. M. (2014). A parent education program for parents of Chinese American children with autism spectrum disorders (ASDS): A pilot study. Focus on Autism and Other Developmental Disabilities, 29(2), 88-94. https://doi.org/doi:https://dx.doi.org/10.1177/1088357613504990

Corti, C., Pergolizzi, F., Vanzin, L., Cargasacchi, G., Villa, L., Pozzi, M., & Molteni, M. (2018). Acceptance and Commitment Therapy-Oriented Parent-Training for Parents of Children with Autism. JOURNAL OF CHILD AND FAMILY STUDIES, 27(9), 2887-2900. https://doi.org/doi:10.1007/s10826-018-1123-3

Coulman, E., Gore, N., Moody, G., Wright, M., Segrott, J., Gillespie, D., Petrou, S., Lugg-Widger, F., Kim, S., Bradshaw, J., McNamara, R., Jahoda, A., Lindsay, G., Shurlock, J., Totsika, V., Stanford, C., Flynn, S., Carter, A., Barlow, C., & Hastings, R. P. (2021). Early Positive Approaches to Support (E-PAtS) for Families of Young Children With Intellectual Disability: A Feasibility Randomised Controlled Trial. FRONTIERS IN PSYCHIATRY, 12. https://doi.org/doi:10.3389/fpsyt.2021.729129

Coulman, E., Hastings, R., Gore, N., Gillespie, D., McNamara, R., Petrou, S., Segrott, J., Bradshaw, J., Hood, K., Jahoda, A., Lindsay, G., Lugg-Widger, F., Robling, M., Shurlock, J., & Totsika, V. (2020). The Early Positive Approaches to Support (E-PAtS) study: study protocol for a feasibility cluster randomised controlled trial of a group programme (E-PAtS) for family caregivers of young children with intellectual disability. PILOT AND FEASIBILITY STUDIES, 6(1). https://doi.org/doi:10.1186/s40814-020-00689-9

Dababnah, S. (2015). Pilot trial of the incredible years for parents of preschool children with autism spectrum disorder. Dissertation Abstracts International Section A: Humanities and Social Sciences, 75(9), No-Specified. https://doi.org/doi:

Dababnah, S., & Parish, S. L. (2016). Incredible Years program tailored to parents of preschoolers with autism: Pilot results. Research on Social Work Practice, 26(4), 372-385. https://doi.org/doi:https://dx.doi.org/10.1177/1049731514558004

Dababnah, S., Kim, I., Magaña, S., & Zhu, Y. (2023). Parents taking action adapted to parents of Black autistic children: Pilot results [Article]. Journal of Policy and Practice in Intellectual Disabilities, 20(1), 18-32. https://doi.org/10.1111/jppi.12423

DaWalt, L. S., Greenberg, J. S., & Mailick, M. R. (2018). Transitioning Together: A Multi-family Group Psychoeducation Program for Adolescents with ASD and Their Parents. JOURNAL OF AUTISM AND DEVELOPMENTAL DISORDERS, 48(1), 251-263. https://doi.org/doi:https://dx.doi.org/10.1007/s10803-017-3307-x

Derguy, C., Poumeyreau, M., Pingault, S., & M'Bailara, K. (2018). [A therapeutic education program for parents of children with ASD: Preliminary results about the effectiveness of the ETAP program]. Un programme d'education therapeutique destine a des parents d'enfant avec un TSA : resultats preliminaires concernant l'efficacite du programme ETAP., 44(5), 421-428. https://doi.org/doi:https://dx.doi.org/10.1016/j.encep.2017.07.004

Dew, A., Collings, S., Dowse, L., Meltzer, A., & Smith, L. (2019). 'I don't feel like I'm in this on my own': Peer support for mothers of children with intellectual disability and challenging behaviour. Journal of intellectual disabilities : JOID, 23(3), 344-358. https://doi.org/doi:https://dx.doi.org/10.1177/1744629519843012

Doenyas, C., & Shohieb, S. M. (2021). Leveraging Technology for the Wellbeing of Individuals With Autism Spectrum Disorder and Their Families During Covid-19. FRONTIERS IN PSYCHIATRY, 12. https://doi.org/doi:10.3389/fpsyt.2021.566809

Duffy, L. V. (2013). Testing the efficacy of the creating opportunities for parent empowerment (COPE) intervention during hospital to home transition: Empowering parents of children with epilepsy and other neurological conditions Boston College]. https://search.ebscohost.com/login.aspx?direct=true&AuthType=ip,uid&db=cin20&AN=109860789&site=ehost-live&scope=site

Dutta, S. (2022). The effects of WHO caregiver skills training on psychological well-being and depression of parents of children with ASD. Developmental Medicine and Child Neurology, 64, 53. https://doi.org/doi:https://dx.doi.org/10.1111/dmcn.15123

Ede, M. O., Anyanwu, J. I., Onuigbo, L. N., Ifelunni, C. O., Alabi-Oparaocha, F. C., Okenyi, E. C., Agu, M. A., Ugwuanyi, L. T., Ugwuanyi, C., Eseadi, C., Awoke, N. N., Nweze, T., & Victor-Aigbodion, V. (2020). Rational Emotive Family Health Therapy for Reducing Parenting Stress in Families of Children with Autism Spectrum Disorders: A Group Randomized Control Study. JOURNAL OF RATIONAL-EMOTIVE AND COGNITIVE-BEHAVIOR THERAPY, 38(2), 243-271. https://doi.org/doi:10.1007/s10942-020-00342-7

Entezami, S., & Souri, A. (2018). Life Skills Training on Reducing Depression and Increasing the Self-esteem of Mentally Disabled Children's Mother. REVISTA PUBLICANDO, 5(15), 367-382. https://doi.org/doi:

Epstein, B. J. (2011). Effects of a Mindfulness-Based Stress Reduction program on fathers of children with developmental disability. Dissertation Abstracts International: Section B: The Sciences and Engineering, 71(10), 6458. https://doi.org/doi:

Erguner-Tekinalp, B., & Akkok, F. (2004). The effects of a coping skills training program on the coping skills, hopelessness, and stress levels of mothers of children with autism. INTERNATIONAL JOURNAL FOR THE ADVANCEMENT OF COUNSELLING, 26(3), 257-269. https://doi.org/doi:https://dx.doi.org/10.1023/B:ADCO.0000035529.92256.0d

Evaluating a group for young people who have a sibling with a disability. (2018). Learning Disability Practice (2014+), 21(3), 17-23. https://doi.org/doi:https://doi.org/10.7748/ldp.2018.e1901

Feinberg, E., Augustyn, M., Fitzgerald, E., Sandler, J., Suarez, Z. F. C., Chen, N., Cabral, H., Beardslee, W., & Silverstein, M. (2014). Improving Maternal Mental Health After a Child's Diagnosis of Autism Spectrum Disorder Results From a Randomized Clinical Trial. JAMA PEDIATRICS, 168(1), 40-46. https://doi.org/doi:10.1001/jamapediatrics.2013.3445

Feinberg, E., Silverstein, M., & Ferreira-Cesar, Z. (2013). Integrating mental health services for mothers of children with autism. Psychiatric Services, 64(9), 930. https://doi.org/doi:https://dx.doi.org/10.1176/appi.ps.640603

Fell, L., Goshe, B., Traeger, L., Perez, G., Iannuzzi, D., Park, E., Kuhlthau, K., & Luberto, C. (2022). Acceptability of A Virtual Mind-Body Group Intervention for Teen Siblings of Children with Autism Spectrum Disorder. JOURNAL OF AUTISM AND DEVELOPMENTAL DISORDERS, 52(12), 5243-5252. https://doi.org/doi:https://dx.doi.org/10.1007/s10803-022-05500-7

Fenton, D. (2019). A centralized internet-based resource center for primary caregivers of children with developmental disabilities. Dissertation Abstracts International: Section B: The Sciences and Engineering, 80(11), No-Specified. https://doi.org/doi:

Fernández-ávalos, M. I., Pérez-Marfil, M. N., Cruz-Quintana, F., Fernández-Alcántara, M., & Ferrer-Cascales, R. (2023). PSYCHOLOGICAL INTERVENTION FOR GRIEF IN CAREGIVERS OF ADULTS DIAGNOSED WITH INTELLECTUAL DISABILITIES [Article]. Behavioral Psychology/ Psicologia Conductual, 31(2), 321-341. https://doi.org/10.51668/bp.8323205n

Ferraioli, S. J., & Harris, S. L. (2013). Comparative effects of mindfulness and skills-based parent training programs for parents of children with autism: Feasibility and preliminary outcome data. Special Issue: Mindfulness and acceptance in developmental disabilities, 4(2), 89-101. https://doi.org/doi:https://dx.doi.org/10.1007/s12671-012-0099-0

Finn, L. L. (2020). Improving quality of life through caregiver training and support. International Journal of Developmental Disabilities, 66(5), 327-329. https://doi.org/doi:10.1080/20473869.2020.1829860

Flujas‑Contreras, J. M., García‑Palacios, A., Castilla, D., & Gómez, I. (2023). Internet‑based versus face‑to‑face Acceptance and Commitment Therapy for parental psychological flexibility.

Flujas-Contreras, J. M., Gomez, I., Garcia-Palacios, A., Chavez, A., & Chavez-Askins, M. (2021). Psychological flexibility and emotional regulation clinical protocol with contextual therapy in families: A case series. Protocolo de intervencion en flexibilidad psicologica y regulacion emocional con terapia contextual en familias: Una serie de casos., 8(3), 50-58. https://doi.org/doi:https://dx.doi.org/10.21134/rpcna.2021.08.3.6

Fuller-Tyszkiewicz, M., Richardson, B., Little, K., Teague, S., Hartley-Clark, L., Capic, T., Khor, S., Cummins, R. A., Olsson, C. A., & Hutchinson, D. (2020). Efficacy of a Smartphone App Intervention for Reducing Caregiver Stress: Randomized Controlled Trial. JMIR MENTAL HEALTH, 7(7), e17541. https://doi.org/doi:https://dx.doi.org/10.2196/17541

Fung, S. C., & Chan, R. C. H. (2024). Evaluation of a person-centred psychotherapy programme for children with developmental disabilities and parents in disadvantaged families [Article]. Counselling and Psychotherapy Research, 24(1), 230-240. https://doi.org/10.1002/capr.12638

Fung, S. C., & Chan, R. C. H. Evaluation of a person-centred psychotherapy programme for children with developmental disabilities and parents in disadvantaged families. COUNSELLING & PSYCHOTHERAPY RESEARCH. https://doi.org/doi:10.1002/capr.12638

Gandia-Abellan, H., Nieto, C., & Marquez-Gonzalez, M. (2020). Working on meaningfulness: Efficacy of a pilot program to improve psychological well-being of parents of children with autism spectrum disorders. Interviniendo en significatividad: Eficacia de un programa piloto en la mejora del bienestar emocional de padres de ninos con trastorno del espectro autista., 28(2), 209-226. https://doi.org/doi:

Gentile, M., Messineo, L., La Guardia, D., Arrigo, M., Citta, G., Ayala, A., Cusimano, G., Martines, P., Mendolia, G., & Allegra, M. (2022). A Parent-Mediated Telehealth Program for Children with Autism Spectrum Disorder : Promoting Parents' Ability to Stimulate the Children's Learning, Reduce Parenting Stress, and Boost Their Sense of Parenting Empowerment. JOURNAL OF AUTISM AND DEVELOPMENTAL DISORDERS, 52(12), 5285-5300. https://doi.org/doi:https://dx.doi.org/10.1007/s10803-022-05482-6

Gillberg, N., Miniscalco, C., & Andersson, G. W. (2023). Knowledge and belongingness: experiences of a programme for parents of children with autism. EDUCATIONAL RESEARCH. https://doi.org/10.1080/00131881.2023.2276956

Gonzalez-Fraile, E., Dominguez-Panchon, A. I., Berzosa, P., Costas-Gonzalez, A. B., Garrido-Jimenez, I., Rufino-Ventura, D., Lopez-Aparicio, J. I., & Martin-Carrasco, M. (2019). Efficacy of a psychoeducational intervention in caregivers of people with intellectual disabilities: A randomized controlled trial (EDUCA-IV trial). RESEARCH IN DEVELOPMENTAL DISABILITIES, 94, 103458. https://doi.org/doi:https://dx.doi.org/10.1016/j.ridd.2019.103458

Gonzalez-Fraile, E., Dominguez-Panchon, A. I., Berzosa, P., Costas-Gonzalez, A. B., Garrido-Jimenez, I., Rufino-Ventura, D., Lopez-Aparicio, J. I., Martin-Carrasco, M., Ballesteros, S. J., Campos, Y. M., Pulido, M. A. B., Mateos, E. N., Martinez, M. D. M., Gonzalez, A. I. P., Velazco, M. D. I., Rodriguez, I. M., Hernandez, C. G., Alfonso, J. C., Gonzalez, N. H., . . . Grp, E.-I. (2019). Efficacy of a psychoeducational intervention in caregivers of people with intellectual disabilities: A randomized controlled trial (EDUCA-IV trial). RESEARCH IN DEVELOPMENTAL DISABILITIES, 94. https://doi.org/doi:10.1016/j.ridd.2019.103458

Gordon, B. K., & Bila, N. J. (2023). Developing a psychoeducational programme for caregivers of people with intellectual disability. AFRICAN JOURNAL OF DISABILITY, 12. https://doi.org/10.4102/ajod.v12i0.1195

Gore, N., Bradshaw, J., Hastings, R., Sweeney, J., & Austin, D. (2022). Early positive approaches to support (E-PAtS): Qualitative experiences of a new support programme for family caregivers of young children with intellectual and developmental disabilities. JOURNAL OF APPLIED RESEARCH IN INTELLECTUAL DISABILITIES, 35(3), 889-899. https://doi.org/doi:10.1111/jar.12993

Greaves, D. (1997). The effect of rational-emotive parent education on the stress of mothers of young children with Down syndrome. Journal of Rational-Emotive & Cognitive-Behavior Therapy, 15(4), 249-267. https://doi.org/doi:https://dx.doi.org/10.1023/A:1025063425172

Grodberg, D., & Smith, I. (2022). Scaling parent management training through digital and microlearning approaches. FRONTIERS IN PSYCHOLOGY, 13. https://doi.org/doi:10.3389/fpsyg.2022.934665

Hahs, A. D., Dixon, M. R., & Paliliunas, D. (2019). Randomized controlled trial of a brief acceptance and commitment training for parents of individuals diagnosed with autism spectrum disorders. JOURNAL OF CONTEXTUAL BEHAVIORAL SCIENCE, 12, 154-159. https://doi.org/doi:10.1016/j.jcbs.2018.03.002

Harris, V., Bourke-Taylor, H. M., & Leo, M. (2022). Healthy Mothers Healthy Families, Health Promoting Activity Coaching for mothers of children with a disability: Exploring mothers' perspectives of programme feasibility. AUSTRALIAN OCCUPATIONAL THERAPY JOURNAL, 69(6), 662-675. https://doi.org/doi:10.1111/1440-1630.12814

Hartley, M., Dorstyn, D., & Due, C. (2022). Challenges encountered with a mindfulness app: Lessons learnt from a pilot randomized trial involving caregivers and individuals with autism.

He, L. H. (2017). Cognitive Behavior Therapy for Asian American Families of Children With Developmental Disabilities. FAMILY JOURNAL, 25(2), 179-186. https://doi.org/doi:10.1177/1066480717697687

Hodes, M. W., Meppelder, M., de Moor, M., Kef, S., & Schuengel, C. (2017). Alleviating Parenting Stress in Parents with Intellectual Disabilities: A Randomized Controlled Trial of a Video-feedback Intervention to Promote Positive Parenting. JOURNAL OF APPLIED RESEARCH IN INTELLECTUAL DISABILITIES, 30(3), 423-432. https://doi.org/doi:10.1111/jar.12302

Hosley, S. N. S. (2022). Challenges to Telehealth: What Was Learned from Families of Children with Neurodevelopmental Disorders. Nursing Clinics of North America, 57(3), 315-328. https://doi.org/doi:10.1016/j.cnur.2022.04.001

Hu, J., Lin, J.-D., Yen, C.-F., Loh, C.-H., Hsu, S.-W., Lin, L.-P., & Wu, S.-R. (2010). Effectiveness of a stress-relief initiative for primary caregivers of adolescents with intellectual disability. JOURNAL OF INTELLECTUAL & DEVELOPMENTAL DISABILITY, 35(1), 29-35. https://doi.org/doi:https://dx.doi.org/10.3109/13668250903501499

Hudson, A., Cameron, C., & Matthews, J. (2008). The wide-scale implementation of a support program for parents of children with an intellectual disability and difficult behaviour. JOURNAL OF INTELLECTUAL & DEVELOPMENTAL DISABILITY, 33(2), 117-126. https://doi.org/doi:https://dx.doi.org/10.1080/13668250802065885

Ibanez, L. V., Kobak, K., Swanson, A., Wallace, L., Warren, Z., & Stone, W. L. (2018). Enhancing interactions during daily routines: A randomized controlled trial of a web-based tutorial for parents of young children with ASD. AUTISM RESEARCH, 11(4), 667-678. https://doi.org/doi:10.1002/aur.1919

Iida, N., Wada, Y., Yamashita, T., Aoyama, M., Hirai, K., & Narumoto, J. (2018). Effectiveness of parent training in improving stress-coping capability, anxiety, and depression in mothers raising children with autism spectrum disorder. Neuropsychiatric disease and treatment, 14, 3355-3362. https://doi.org/doi:https://dx.doi.org/10.2147/NDT.S188387

Inoue, M., Inoue, N., Nakatani, K., & Shikibu, Y. (2023). Online Parent Training for Parents of Children with Autism Spectrum Disorders: Prototype Development of the On-Demand Type [Article]. Yonago Acta Medica, 66(1), 95-103. https://doi.org/10.33160/yam.2023.02.012

Intagliata, J., & Doyle, N. (1984). Enhancing social support for parents of developmentally disabled children: Training in interpersonal problem solving skills. Mental Retardation, 22(1), 4-11. https://doi.org/doi:

Jalali, L. C., Hasanzadeh, S., Davaee, M., & Afrooz, G. (2016). Development and assessment of effects of de-stress training program on stress of mothers with mentally disabled children. Iranian Rehabilitation Journal, 14(4), 223-228. https://doi.org/doi:10.18869/nrip.irj.14.4.223

Jenaabadi, H., & Abbasi, N. (2020). Effectiveness of Group Training of Reliance Therapy Components on Quality of Life and Resilience of Mothers of Children with Learning Disabilities. Health, Spirituality & Medical Ethics Journal, 7(1), 25-31. https://doi.org/doi:10.29252/jhsme.7.1.25

Jones, L., Gold, E., Totsika, V., Hastings, R. P., Jones, M., Griffiths, A., & Silverton, S. (2018). A mindfulness parent well-being course: evaluation of outcomes for parents of children with autism and related disabilities recruited through special schools. EUROPEAN JOURNAL OF SPECIAL NEEDS EDUCATION, 33(1), 16-30. https://doi.org/doi:10.1080/08856257.2017.1297571

Kaçan, H., Gümüs, F., & Deger, V. B. (2023). Effect of individual psychoeducation for primary caregivers of children with autism on internalized stigma and care burden: a randomized controlled trial. INTERNATIONAL JOURNAL OF DEVELOPMENTAL DISABILITIES. https://doi.org/10.1080/20473869.2023.2231233

Kakabaraee, K., & Seidy, M. (2021). Effect of the Problem-solving on the Mental Well-being of Mothers With Autistic Children. Journal of Research and Health, 11(3), 203-212. https://doi.org/doi:10.32598/JRH.11.3.118.4

Kalantary, M. R., Donyavi, R., & Rostamian, M. (2019). Effectiveness of positive psychotherapy on perceived stress and the reasons for living in mothers of children with autism. Journal of Mazandaran University of Medical Sciences, 29(180), 86-94. https://doi.org/doi:

Kaltenbach, E., Chisholm, M., Xiong, T., Thomson, D., Crombach, A., & McGrath, P. J. (2021). Online narrative exposure therapy for parents of children with neurodevelopmental disabilities suffering from posttraumatic stress symptoms–study protocol of a randomized controlled trial. European Journal of Psychotraumatology, 12(1). https://doi.org/doi:10.1080/20008198.2021.1991650

Kang, S. J., Kim, H. S., & Baek, K. H. (2021). Effects of Nature-Based Group Art Therapy Programs on Stress, Self-Esteem and Changes in Electroencephalogram (EEG) in Non-Disabled Siblings of Children with Disabilities. INTERNATIONAL JOURNAL OF ENVIRONMENTAL RESEARCH AND PUBLIC HEALTH, 18(11). https://doi.org/doi:10.3390/ijerph18115912

Karakurt, N., Durmaz, H., & Gürol, A. (2023). The Effect of Mindfulness-Based Training on the Level of Depression in Mothers of Children with Down Syndrome. AMERICAN JOURNAL OF FAMILY THERAPY. https://doi.org/10.1080/01926187.2023.2282975

Karp, E. A., & Kuo, A. A. (2015). Maternal Mental Health After a Child's Diagnosis of Autism Spectrum Disorder. JAMA-JOURNAL OF THE AMERICAN MEDICAL ASSOCIATION, 313(1), 81-+. https://doi.org/doi:10.1001/jama.2014.11187

Kawada, M., & Nojima, S. (2020). Evaluating the Effects of the Empowerment Program for Parents of Adults with High-Functioning Autistic Spectrum Disorder. Journal of Psychosocial Rehabilitation and Mental Health, 7(1), 27-44. https://doi.org/doi:10.1007/s40737-020-00155-y

Ketcheson, L. R., Wengrovius, C. M., Staples, K. L., & Miodrag, N. (2022). MYTime: A Mindfulness and Yoga Program to Promote Health Outcomes in Parents of Children With Autism Spectrum Disorder. Global Advances in Health and Medicine, 11, 2164957X221110154. https://doi.org/doi:https://dx.doi.org/10.1177/2164957X221110154

Khorramabadi, R., Pouretemad, H., Dehghani, M., & Mazaheri, M. (2009). THE EFFECTIVENESS OF LITTLE BIRD PROGRAMME ON IMPROVEMENT OF MENTAL HEALTH AND DECREASE PARENTAL STRESS IN MOTHERS OF AUTISTIC CHILDREN. EUROPEAN PSYCHIATRY, 24. https://doi.org/doi:

Kramer, J. M., Beasley, J. B., Caoili, A., Goode, T., Guerrero, F., Klein, A., Grosso, E., & Kennelly-Smith, E. (2023). Caregiver Experiences with Teleservices for People with Intellectual and Developmental Disabilities and Mental Health Needs During the Onset of COVID-19 [Article]. Journal of Mental Health Research in Intellectual Disabilities, 16(3), 186-204. https://doi.org/10.1080/19315864.2023.2214096

Krstic, T., Mihic, I., & Brankovic, J. (2021). "Our Story": Exposition of a Group Support Program for Parents of Children with Developmental Disabilities. Child Care in Practice, 27(4), 406-421. https://doi.org/doi:https://dx.doi.org/10.1080/13575279.2019.1664991

Kryzak, L. A., Cengher, M., Feeley, K. M., Fienup, D. M., & Jones, E. A. (2015). A community support program for children with autism and their typically developing siblings: Initial investigation. Journal of intellectual disabilities : JOID, 19(2), 159-177. https://doi.org/doi:https://dx.doi.org/10.1177/1744629514564450

Kuhlthau, K. A., Traeger, L., Luberto, C. M., Perez, G. K., Goshe, B. M., Fell, L., Iannuzzi, D., & Park, E. R. (2022). Resiliency Intervention for Siblings of Children With Autism Spectrum Disorder: A Randomized Pilot Trial. Academic pediatrics. https://doi.org/doi:https://dx.doi.org/10.1016/j.acap.2022.11.011

Lappalainen, P., Gallego, A., Keinonen, K., Lappalainen, A.-L., Tolvanen, A., & Lappalainen, R. (2024). Online and Self-Help Acceptance and Commitment Therapy for Parents of Children with Chronic Conditions and Developmental Disabilities: What Happens after the Intervention? Child & Family Behavior Therapy, 46(1), 1-26. https://doi.org/10.1080/07317107.2023.2233949

Lappalainen, P., Pakkala, I., Strömmer, J., Sairanen, E., Kaipainen, K., & Lappalainen, R. (2021). Supporting parents of children with chronic conditions: A randomized controlled trial of web-based and self-help ACT interventions. Internet Interventions, 24. https://doi.org/doi:10.1016/j.invent.2021.100382

Larson, E. A. (2023). 5Minutes4Myself: Development of a Wellness Program for Caregivers of Children with Autism. Canadian journal of occupational therapy. Revue canadienne d'ergotherapie, 84174231172036. https://doi.org/doi:https://dx.doi.org/10.1177/00084174231172036

Larson, E. A. (2023). 5Minutes4Myself: Development of a Wellness Program for Caregivers of Children with Autism [Article]. Canadian Journal of Occupational Therapy. https://doi.org/10.1177/00084174231172036

Larson, E. A. 5Minutes4Myself: Development of a Wellness Program for Caregivers of Children with Autism. CANADIAN JOURNAL OF OCCUPATIONAL THERAPY-REVUE CANADIENNE D ERGOTHERAPIE. https://doi.org/doi:10.1177/00084174231172036

Larson, E., Jeglum, N., & Shmays, C. (2023). Mixed-method evaluation of fidelity of motivational interviewing-based coaching in 5Minutes4Myself Wellness Program for caregivers of children with autism [Article]. PEC Innovation, 2. https://doi.org/10.1016/j.pecinn.2023.100149

Lau, J. S. P., Lai, S. M. K., Ip, F. T. S., Wong, P. W. C., Team, W. C., Servili, C., Salomone, E., Pacione, L., Shire, S., & Brown, F. L. (2022). Acceptability and feasibility of the World Health Organization's Caregiver Skills Training Programme (WHO CST) delivered via eLearning, videoconferencing, and in-person hybrid modalities in Hong Kong. FRONTIERS IN PSYCHIATRY, 13. https://doi.org/doi:10.3389/fpsyt.2022.915263

Lau, J. S.-P., Lai, S. M.-K., Ip, F. T.-S., Wong, P. W.-C., Team, W. C., Servili, C., Salomone, E., Pacione, L., Shire, S., & Brown, F. L. (2022). Acceptability and feasibility of the World Health Organization's Caregiver Skills Training Programme (WHO CST) delivered via eLearning, videoconferencing, and in-person hybrid modalities in Hong Kong. FRONTIERS IN PSYCHIATRY, 13, 915263. https://doi.org/doi:https://dx.doi.org/10.3389/fpsyt.2022.915263

Leadbitter, K., Smallman, R., James, K., Shields, G., Ellis, C., Langhorne, S., Harrison, L., Hackett, L., Dunkerley, A., Kroll, L., Davies, L., Emsley, R., Bee, P., Green, J., & Team, R. A. (2022). REACH-ASD: a UK randomised controlled trial of a new post-diagnostic psycho-education and acceptance and commitment therapy programme against treatment-as-usual for improving the mental health and adjustment of caregivers of children recently diagnosed with autism spectrum disorder. TRIALS, 23(1). https://doi.org/doi:10.1186/s13063-022-06524-1

Leadbitter, K., Smallman, R., James, K., Shields, G., Ellis, C., Langhorne, S., Harrison, L., Hackett, L., Dunkerley, A., Kroll, L., Davies, L., Emsley, R., Bee, P., Green, J., The, R.-A. S. D. T., Ahmed, S., Beach, H., Butter, C., Gilbert, J., & Goldie, C. (2022). REACH-ASD: a UK randomised controlled trial of a new post-diagnostic psycho-education and acceptance and commitment therapy programme against treatment-as-usual for improving the mental health and adjustment of caregivers of children recently diagnosed with autism spectrum disorder. TRIALS, 23(1), 1-18. https://doi.org/doi:10.1186/s13063-022-06524-1

Lindsey, J. G. (2021). Program evaluation of a rural support group for parents of children with autism. Dissertation Abstracts International: Section B: The Sciences and Engineering, 82(8), No-Specified. https://doi.org/doi:

Little, L. M., Wallisch, A., Pope, E., & Dunn, W. (2018). Acceptability and Cost Comparison of a Telehealth Intervention for Families of Children With Autism. INFANTS & YOUNG CHILDREN, 31(4), 275-286. https://doi.org/doi:10.1097/IYC.0000000000000126

Liu, G. H., Wang, S., Liao, J. H., Ou, P., Huang, L. S., Xie, N. M., He, Y. S., Lin, J. L., He, H. G., & Hu, R. F. (2021). The Efficacy of WeChat-Based Parenting Training on the Psychological Well-being of Mothers With Children With Autism During the COVID-19 Pandemic: Quasi-Experimental Study. JMIR MENTAL HEALTH, 8(2). https://doi.org/doi:10.2196/23917

Liu, G., Wang, S., Liao, J., Ou, P., Huang, L., Xie, N., He, Y., Lin, J., He, H.-G., & Hu, R. (2021). The Efficacy of WeChat-Based Parenting Training on the Psychological Well-being of Mothers With Children With Autism During the COVID-19 Pandemic: Quasi-Experimental Study. JMIR MENTAL HEALTH, 8(2), e23917. https://doi.org/doi:https://dx.doi.org/10.2196/23917

Lo Julia Wing, K., Ma Joyce Lai, C., Wong Mooly Mei, C., & Yau-Ng Monica Lai, T. (2023). Virtual care during the pandemic: Multi-family group sessions for Hong Kong Chinese families of adolescents with intellectual disabilities. Journal of Intellectual Disabilities, 27(2), 336-353. https://doi.org/https://doi.org/10.1177/17446295221076693

Lo, H. H. M., Chan, S. K. C., Szeto, M. P., Chan, C. Y. H., & Choi, C. W. (2017). A feasibility study of a brief mindfulness-based program for parents of preschool children with developmental disabilities. Mindfulness, 8(6), 1665-1673. https://doi.org/doi:https://dx.doi.org/10.1007/s12671-017-0741-y

Lo, T. L. T., Wan, A. H. Y., Fong, T. C. T., Wong, P. K. S., Lo, H. H. M., Chan, C. K. P., & Ho, R. T. H. (2023). Protocol for a mixed-methods randomised controlled trial evaluating the effectiveness of a dyadic expressive arts-based intervention in improving the psychosocial well-being of children with intellectual disability in special schools and their mothers. BMJ OPEN, 13(7). https://doi.org/10.1136/bmjopen-2022-067239

Lobato, D., Montesinos, F., Polin, E., & Caliz, S. (2022). Acceptance and Commitment Training Focused on Psychological Flexibility for Family Members of Children with Intellectual Disabilities. INTERNATIONAL JOURNAL OF ENVIRONMENTAL RESEARCH AND PUBLIC HEALTH, 19(21). https://doi.org/doi:10.3390/ijerph192113943

Lobato, D., Montesinos, F., Polín, E., & Cáliz, S. (2023). Third-Generation Behavioural Therapies in the Context of Neurodevelopmental Problems and Intellectual Disabilities: A Randomised Clinical Trial with Parents. INTERNATIONAL JOURNAL OF ENVIRONMENTAL RESEARCH AND PUBLIC HEALTH, 20(5). https://doi.org/doi:10.3390/ijerph20054406

Lobato, D., Montesinos, F., Polín, E., & Cáliz, S. (2023). Third-Generation Behavioural Therapies in the Context of Neurodevelopmental Problems and Intellectual Disabilities: A Randomised Clinical Trial with Parents [Article]. International Journal of Environmental Research and Public Health, 20(5). https://doi.org/10.3390/ijerph20054406

Lodder, A., Papadopoulos, C., & Randhawa, G. (2020). SOLACE: A Psychosocial Stigma Protection Intervention to Improve the Mental Health of Parents of Autistic Children—A Feasibility Randomised Controlled Trial. JOURNAL OF AUTISM AND DEVELOPMENTAL DISORDERS, 50(12), 4477-4491. https://doi.org/doi:10.1007/s10803-020-04498-0

Lodder, A., Papadopoulos, C., & Randhawa, G. (2020). Using a blended format (videoconference and face to face) to deliver a group psychosocial intervention to parents of autistic children (Vol. 21). Elsevier B.V. https://doi.org/doi:10.1016/j.invent.2020.100336

Lopez-Liria, R., Vargas-Munoz, E., Aguilar-Parra, J. M., Padilla-Gongora, D., Manas-Rodriguez, M. A., & Rocamora-Perez, P. (2020). Effectiveness of a Training Program in the Management of Stress for Parents of Disabled Children. JOURNAL OF CHILD AND FAMILY STUDIES, 29(4), 964-977. https://doi.org/doi:10.1007/s10826-019-01665-5

Magana, S., Li, H., Miranda, E., & de Sayu, R. P. (2015). Improving health behaviours of Latina mothers of youths and adults with intellectual and developmental disabilities. JOURNAL OF INTELLECTUAL DISABILITY RESEARCH, 59(5), 397-410. https://doi.org/doi:10.1111/jir.12139

Magana, S., Tejero Hughes, M., Salkas, K., Gonzales, W., Nunez, G., Morales, M., Garcia Torres, M., & Moreno-Angarita, M. (2021). Implementing a Parent Education Intervention in Colombia: Assessing Parent Outcomes and Perceptions Across Delivery Modes. Focus on Autism and Other Developmental Disabilities, 36(3), 165-175. https://doi.org/doi:https://dx.doi.org/10.1177/1088357620986947

Mai, T. L. A., & Chaimongkol, N. (2022). Effectiveness of a Family Management Intervention Program among Families of Children with Autism: A Randomized Controlled Trial. PACIFIC RIM INTERNATIONAL JOURNAL OF NURSING RESEARCH, 26(1), 63-77. https://doi.org/doi:

Marino, L. J. (1951). Organizing the parents of mentally retarded children for participation in the mental-health program. Mental hygiene, 35(1), 14-18. https://doi.org/doi:

Martin, F., Clyne, W., Pearce, G., & Turner, A. (2019). Self-Management Support Intervention for Parents of Children with Developmental Disorders: The Role of Gratitude and Hope. JOURNAL OF CHILD AND FAMILY STUDIES, 28(4), 980-992. https://doi.org/doi:10.1007/s10826-018-01308-1

Martino, E. M. (2013). Group effort: educational support groups for parents of children with autism spectrum disorder. OT Practice, 18(2), 14-18. https://doi.org/doi:

Mason, R. (2005). Person-centred approaches for parents -- a mother's view. Living Well, 5(3), 12-15. https://doi.org/doi:

Maughan, A. L., Lunsky, Y., Lake, J., Mills, J. S., Fung, K., Steel, L., & Weiss, J. A. (2023). Parent, child, and family outcomes following Acceptance And Commitment Therapy for parents of autistic children: A randomized controlled trial [Article]. Autism. https://doi.org/10.1177/13623613231172241

May, C. D., St George, J. M., & Lane, S. (2021). From presence to participation: Engagement with an SMS program for fathers of children on the autism spectrum. JOURNAL OF CHILD AND FAMILY STUDIES, 30(1), 29-37. https://doi.org/doi:https://dx.doi.org/10.1007/s10826-020-01845-8

May, C. D., St George, J. M., & Lane, S. (2022). Fathers Raising Children on the Autism Spectrum: Lower Stress and Higher Self-Efficacy Following SMS (Text2dads) Intervention. JOURNAL OF AUTISM AND DEVELOPMENTAL DISORDERS, 52(1), 306-315. https://doi.org/doi:https://dx.doi.org/10.1007/s10803-021-04925-w

May, C. D., St George, J. M., & Lane, S. (2022). Fathers raising children on the autism spectrum: Lower stress and higher self-efficacy following SMS (Text2dads) intervention.

May, F. S., McLean, L. A., Anderson, A., Hudson, A., Cameron, C., & Matthews, J. (2013). Father participation with mothers in the Signposts program: an initial investigation. JOURNAL OF INTELLECTUAL & DEVELOPMENTAL DISABILITY, 38(1), 39-47. https://doi.org/doi:https://dx.doi.org/10.3109/13668250.2012.748184

Mevissen, L., Ooms-Evers, M., Serra, M., de Jongh, A., & Didden, R. (2020). Intensive trauma focused treatment for families with mild intellectual disability: Feasibility and potential effectiveness. Intensieve traumagerichte behandeling voor gezinnen met LVB: Toepasbaarheid en potentiele effectiviteit., 53(4), 237-256. https://doi.org/doi:

Milberger, S., Marsack-Topolewski, C., Janks, E., Anderson, N., Bray, M., & Samuel, P. S. (2022). Evaluating the benefits of a family support program on the health and well-being of aging family caregivers of adults with intellectual and developmental disabilities. Journal of Gerontological Social Work, No-Specified. https://doi.org/doi:https://dx.doi.org/10.1080/01634372.2022.2110347

Milberger, S., Marsack-Topolewski, C., Janks, E., Anderson, N., Bray, M., & Samuel, P. S. (2023). Evaluating the Benefits of a Family Support Program on the Health and Well-Being of Aging Family Caregivers of Adults with Intellectual and Developmental Disabilities. Journal of Gerontological Social Work, 66(3), 413-432. https://doi.org/doi:https://dx.doi.org/10.1080/01634372.2022.2110347

Milberger, S., Marsack-Topolewski, C., Janks, E., Anderson, N., Bray, M., & Samuel, P. S. (2023). Evaluating the Benefits of a Family Support Program on the Health and Well-Being of Aging Family Caregivers of Adults with Intellectual and Developmental Disabilities [Article]. Journal of Gerontological Social Work, 66(3), 413-432. https://doi.org/10.1080/01634372.2022.2110347

Miller, L., Imms, C., Cross, A., Pozniak, K., O’Connor, B., Martens, R., Cavalieros, V., Babic, R., Novak-Pavlic, M., Rodrigues, M., Balram, A., Hughes, D., Ziviani, J., & Rosenbaum, P. (2023). Impact of “early intervention” parent workshops on outcomes for caregivers of children with neurodisabilities: a mixed-methods study [Article]. Disability and Rehabilitation, 45(23), 3900-3911. https://doi.org/10.1080/09638288.2022.2143579

Mills, A. S., Vimalakanthan, K., Sivapalan, S., Shanmugalingam, N., & Weiss, J. A. (2020). Brief Report: Preliminary Outcomes of a Peer Counselling Program for Parents of Children with Autism in the South Asian Community. JOURNAL OF AUTISM AND DEVELOPMENTAL DISORDERS. https://doi.org/doi:https://dx.doi.org/10.1007/s10803-020-04538-9

Mills, A. S., Vimalakanthan, K., Sivapalan, S., Shanmugalingam, N., & Weiss, J. A. (2021). Brief Report: Preliminary Outcomes of a Peer Counselling Program for Parents of Children with Autism in the South Asian Community. JOURNAL OF AUTISM AND DEVELOPMENTAL DISORDERS, 51(1), 334-340. https://doi.org/doi:https://dx.doi.org/10.1007/s10803-020-04538-9

Millstein, R. A., Lindly, O. J., Luberto, C. M., Perez, G. K., Schwartz, G. N., Kuhlthau, K., & Park, E. R. (2020). An Exploration of Health Behaviors in a Mind-Body Resilience Intervention for Parents of Children with Developmental Disabilities. Journal of developmental and behavioral pediatrics : JDBP, 41(6), 480-485. https://doi.org/doi:https://dx.doi.org/10.1097/DBP.0000000000000813

Miodrag, N., & Dykens, E. M. (2012). MBSR: Mental health outcomes for parents of children with autism spectrum disorders and other I/DD conditions. JOURNAL OF INTELLECTUAL DISABILITY RESEARCH, 56(7), 737. https://doi.org/doi:https://dx.doi.org/10.1111/j.1365-2788.2012.01583_8.x

Muthukaruppan, S. S., Cameron, C., Campbell, Z., Krishna, D., Moineddin, R., Bharathwaj, A., Poomariappan, B. M., Mariappan, S., Boychuk, N., Ponnusamy, R., MacLachlan, J., Brien, M., Nixon, S., & Srinivasan, S. R. (2022). Impact of a family-centred early intervention programme in South India on caregivers of children with developmental delays. Disability & Rehabilitation, 44(11), 2410-2419. https://doi.org/doi:10.1080/09638288.2020.1836046

Naheed, A., Islam, M. S., Brooks, M. B., Fawzi, M. C. S., Ashraf, M. N., Ahmed, H. U., Uddin, M. M. J., Koly, K. N., Galea, J. T., Akhter, S., Nelson, C., Hossain, S. W., & Munir, K. M. (2022). Feasibility of a school-based mental health program implementation to improve the status of depression and quality of life of mothers of children with autism spectrum disorders in urban Bangladesh: MENTHOL study. Global mental health (Cambridge, England), 9, 146-156. https://doi.org/doi:https://dx.doi.org/10.1017/gmh.2022.16

Naheed, A., Koly, K. N., Ahmed, H. U., Akhter, S., Mannan, M., Uddin, M. M. J., Fawzi, M. C. S., Chandir, S., Hossain, S., Nelson, C., & Munir, K. (2017). Implementing a Mental Health Care Program and Home-Based Training for Mothers of Children With Autism Spectrum Disorder in an Urban Population in Bangladesh: Protocol for a Feasibility Assessment Study. Journal of Medical Internet Research, 19(12), 1-1. https://doi.org/doi:10.2196/resprot.8260

Neece, C. L., Fenning, R. M., Morrell, H. E. R., & Benjamin, L. R. (2023). Comparative effects of mindfulness-based stress reduction and psychoeducational support on parenting stress in families of autistic preschoolers. Autism. https://doi.org/10.1177/13623613231191558

Niinomi, K., Asano, M., Kadoma, A., Yoshida, K., Ohashi, Y., Furuzawa, A., Yamamoto, M., Yamakita, N., & Mori, A. (2016). Developing the "Skippu-Mama" program for mothers of children with autism spectrum disorder. Nursing & health sciences, 18(3), 283-291. https://doi.org/doi:https://dx.doi.org/10.1111/nhs.12264

Onyishi, C. N., Sefotho, M. M., & Victor-Aibodion, V. (2023). Psychological distress among parents of children with autism spectrum disorders: A randomized control trial of cognitive behavioural therapy. Research in Autism Spectrum Disorders, 100, 102070. https://doi.org/doi:https://dx.doi.org/10.1016/j.rasd.2022.102070

Onyishi, C. N., Sefotho, M. M., & Victor-Aibodion, V. (2023). Psychological distress among parents of children with autism spectrum disorders: A randomized control trial of cognitive behavioural therapy [Article]. Research in Autism Spectrum Disorders, 100. https://doi.org/10.1016/j.rasd.2022.102070

Park, E. R., Perez, G. K., Millstein, R. A., Luberto, C. M., Traeger, L., Proszynski, J., Chad-Friedman, E., & Kuhlthau, K. A. (2020). A Virtual Resiliency Intervention Promoting Resiliency for Parents of Children with Learning and Attentional Disabilities: A Randomized Pilot Trial. Maternal and Child Health Journal, 24(1), 39-53. https://doi.org/doi:10.1007/s10995-019-02815-3

Paterson, C. (2006). Development of a cognitive-behaviour therapy workbook program for symptoms of stress, anxiety or depression in parents of children with intellectual disability and challenging behaviour. JOURNAL OF INTELLECTUAL & DEVELOPMENTAL DISABILITY, 31(1), 58-58. https://doi.org/doi:10.1080/13668250500488686

Perdomo, J., Hernandez, B., Munoz Ruiz, J., Griffin, M., & Rea, C. J. (2022). Impacting Caregiver Self-efficacy and Stress During the COVID-19 Pandemic Through a Virtual Toddler Group Visit Pilot. Clinical Pediatrics, 61(1), 12-16. https://doi.org/doi:10.1177/00099228211048597

Perry, E. (2022). Special Parenting Workshop - Workplace Intervention. Israel Journal of Occupational Therapy, 31(2), H146-H152. https://doi.org/doi:

Petcharat, M., & Liehr, P. (2021). Feasibility of a brief mindfulness intervention: Examining stress, anxiety and mindfulness for Thai parents of children with developmental disabilities. Archives of psychiatric nursing, 35(5), 418-426. https://doi.org/doi:https://dx.doi.org/10.1016/j.apnu.2021.06.002

Phillips, R. S. C. (1999). Intervention with siblings of children with developmental disabilities from economically disadvantaged families. FAMILIES IN SOCIETY-THE JOURNAL OF CONTEMPORARY HUMAN SERVICES, 80(6), 569-577. https://doi.org/doi:10.1606/1044-3894.1781

Pourmohamadreza-Tajrishi, M., Azadfallah, P., Garakani, S. H., & Bakhshi, E. (2015). The Effect of Problem-Focused Coping Strategy Training on Psychological Symptoms of Mothers of Children with Down Syndrome. IRANIAN JOURNAL OF PUBLIC HEALTH, 44(2), 254-262. https://doi.org/doi:

Pourmohamadreza-Tajrishi, M., Azadfallah, P., Hemmati Garakani, S., & Bakhshi, E. (2015). The effect of problem-focused coping strategy training on psychological symptoms of mothers of children with down syndrome. IRANIAN JOURNAL OF PUBLIC HEALTH, 44(2), 254-262. https://doi.org/doi:

Raulston, T. J., Zemantic, P. K., Machalicek, W., Hieneman, M., Kurtz-Nelson, E., Barton, H., Hansen, S. G., & Frantz, R. J. (2019). Effects of a brief mindfulness-infused behavioral parent training for mothers of children with autism spectrum disorder. JOURNAL OF CONTEXTUAL BEHAVIORAL SCIENCE, 13, 42-51. https://doi.org/doi:https://dx.doi.org/10.1016/j.jcbs.2019.05.001

Rayan, A., & Ahmad, M. (2016). Effectiveness of mindfulness-based interventions on quality of life and positive reappraisal coping among parents of children with autism spectrum disorder. RESEARCH IN DEVELOPMENTAL DISABILITIES, 55, 185-196. https://doi.org/doi:https://dx.doi.org/10.1016/j.ridd.2016.04.002

Rayan, A., & Ahmad, M. (2017). Effectiveness of mindfulness-based intervention on perceived stress, anxiety, and depression among parents of children with autism spectrum disorder. Mindfulness, 8(3), 677-690. https://doi.org/doi:https://dx.doi.org/10.1007/s12671-016-0595-8

Rayan, A., & Ahmad, M. (2018). Psychological Distress in Jordanian Parents of Children With Autism Spectrum Disorder: The Role of Trait Mindfulness. PERSPECTIVES IN PSYCHIATRIC CARE, 54(1), 11-18. https://doi.org/doi:10.1111/ppc.12187

Reid, C., Gill, F., Gore, N., & Brady, S. (2016). New ways of seeing and being: Evaluating an acceptance and mindfulness group for parents of young people with intellectual disabilities who display challenging behaviour. Journal of Intellectual Disabilities, 20(1), 5-17. https://doi.org/doi:10.1177/1744629515584868

Ridderinkhof, A., de Bruin, E. I., Blom, R., Singh, N. N., & Bogels, S. M. (2019). Mindfulness-based program for autism spectrum disorder: A qualitative study of the experiences of children and parents. Mindfulness, 10(9), 1936-1951. https://doi.org/doi:https://dx.doi.org/10.1007/s12671-019-01202-x

Roberts, D. B. R. H. V. R. G. N. R., & Pickering, N. D. B. A. (2010). Parent training programme for autism spectrum disorders: an evaluation: The Journal of the Health Visitors' Association. Community Practitioner, 83(10), 27-30. https://doi.org/doi:

Rojas-Torres, L. P., Alonso-Esteban, Y., & Alcantud-Marín, F. (2023). Mindfulness Parenting and Childish Play: A Clinical Trial With Parents of Children With Autism Spectrum Disorders [Article]. Psicothema, 35(3), 259-270. https://doi.org/10.7334/psicothema2022.339

Rojas-Torres, L. P., Alonso-Esteban, Y., Lopez-Ramon, M. F., & Alcantud-Marin, F. (2021). Mindfulness-Based Stress Reduction (MBSR) and Self Compassion (SC) Training for Parents of Children with Autism Spectrum Disorders: A Pilot Trial in Community Services in Spain. Children (Basel, Switzerland), 8(5). https://doi.org/doi:https://dx.doi.org/10.3390/children8050316

Romero-Martinez, A., Ruiz-Robledillo, N., Sarinana-Gonzalez, P., de Andres-Garcia, S., Vitoria-Estruch, S., & Moya-Albiol, L. (2017). A cognitive-behavioural intervention improves cognition in caregivers of people with autism spectrum disorder: A pilot study. PSYCHOSOCIAL INTERVENTION, 26(3), 165-170. https://doi.org/doi:10.1016/j.psi.2017.06.002

Ruiz-Robledillo, N., & Moya-Albiol, L. (2015). Effects of a cognitive-behavioral intervention program on the health of caregivers of people with autism spectrum disorder. PSYCHOSOCIAL INTERVENTION, 24(1), 33-39. https://doi.org/doi:https://dx.doi.org/10.1016/j.psi.2015.01.001

Ruiz-Robledillo, N., Sarinana-Gonzalez, P., Perez-Blasco, J., Gonzalez-Bono, E., & Moya-Albiol, L. (2015). A mindfulness-based program improves health in caregivers of people with autism spectrum disorder: A pilot study. Mindfulness, 6(4), 767-777. https://doi.org/doi:https://dx.doi.org/10.1007/s12671-014-0316-0

Saba, S., Arsalani, N., Hosseini, M. A., Soltani, P. R., & Azizi, M. (2023). The Effect of Resilience Training on the Stress of Mothers of Students With Down Syndrome [Article]. Iranian Rehabilitation Journal, 21(3), 525-532. https://doi.org/10.32598/irj.21.3.1906.1

Safara, M., Khanbabaee, M., & Khanbabaee, M. (2019). The Effect of Spiritual Skills Training on the Quality of Life in Mothers of Mentally Retarded Children. Health, Spirituality & Medical Ethics Journal, 6(2), 24-30. https://doi.org/doi:10.29252/jhsme.6.2.24

Safer-Lichtenstein, J. (2023). Feasibility and acceptability of parenting interventions delivered in Spanish to caregivers of children with autism and other developmental delays: A mixed-methods design. Dissertation Abstracts International: Section B: The Sciences and Engineering, 84(2), No-Specified. https://doi.org/doi:

Salem-Guirgis, S., Albaum, C., Tablon, P., Riosa, P. B., Nicholas, D. B., Drmic, I. E., & Weiss, J. A. (2019). MYmind: a Concurrent Group-Based Mindfulness Intervention for Youth with Autism and Their Parents. Mindfulness, 10(9), 1730-1743. https://doi.org/doi:https://dx.doi.org/10.1007/s12671-019-01107-9

Sarang, S. D., Karnam, A. G., Vanmali, B. A., & Phulpagar, P. R. (2020). Effect of Mindfulness-Based Stress Reduction Occupational Therapy Program in Parents of Children with Autism Spectrum Disorder: An Interventional Study. Indian Journal of Occupational Therapy (Wolters Kluwer India Pvt Ltd), 52(4), 132-138. https://doi.org/doi:10.4103/ijoth.ijoth_25_20

Schlebusch, L., Chambers, N., Rosenstein, D., Erasmus, P., & de Vries, P. J. (2024). Supporting caregivers of children with developmental disabilities: Findings from a brief caregiver well-being programme in South Africa. Autism: The International Journal of Research & Practice, 28(1), 199-214. https://doi.org/10.1177/13623613221133182

Schwartzman, J. M., Millan, M. E., Uljarevic, M., & Gengoux, G. W. (2022). Resilience Intervention for Parents of Children with Autism: Findings from a Randomized Controlled Trial of the AMOR Method. JOURNAL OF AUTISM AND DEVELOPMENTAL DISORDERS, 52(2), 738-757. https://doi.org/doi:10.1007/s10803-021-04977-y

Shaffer, E. J., Lape, J. E., & Salls, J. (2020). Decreasing Stress for Parents of Special Needs Children through a Web-Based Mindfulness Program: A Pilot Study. INTERNET JOURNAL OF ALLIED HEALTH SCIENCES AND PRACTICE, 18(4). https://doi.org/doi:

Shahrivar, Z., Rabiee, A., & Tehrani Doost, M. (2015). Efficacy of group educational training on knowledge, stress, anxiety and depression in parents of children with autistic disorder. European Child and Adolescent Psychiatry, 24(1), S195. https://doi.org/doi:https://dx.doi.org/10.1007/s00787-015-0714-4

Sherman, J., Larson, E., Newlon, J., & Rowley, M. (2019). A Wellness Tool for Caregivers of Children With Autism Spectrum Disorder: Assessing Usability of the 5Minutes4Myself Companion App. American Journal of Occupational Therapy, 73, 1-1. https://doi.org/doi:10.5014/ajot.2019.73S1-PO3025

Shu, B. C., & Lung, F. W. (2005). The effect of support group on the mental health and quality of life for mothers with autistic children. JOURNAL OF INTELLECTUAL DISABILITY RESEARCH, 49, 47-53. https://doi.org/doi:10.1111/j.1365-2788.2005.00661.x

Shu, B. C., Lung, F. W., & Huang, C. (2002). Mental health of primary family caregivers with children with intellectual disability who receive a home care programme. JOURNAL OF INTELLECTUAL DISABILITY RESEARCH, 46, 257-263. https://doi.org/doi:10.1046/j.1365-2788.2002.00370.x

Shu, B. C., Lung, F. W., Chen, Y. C., & Chen, B. C. (2000). Mental health in family caregivers with children with intellectual disability who receive a home care programme. JOURNAL OF INTELLECTUAL DISABILITY RESEARCH, 44, 463-463. https://doi.org/doi:

Singh, N. N., Lancioni, G. E., Karazsia, B. T., & Myers, R. E. (2016). Caregiver Training in Mindfulness-Based Positive Behavior Supports (MBPBS): Effects on Caregivers and Adults with Intellectual and Developmental Disabilities. FRONTIERS IN PSYCHOLOGY, 7, 98. https://doi.org/doi:https://dx.doi.org/10.3389/fpsyg.2016.00098

Singh, N. N., Lancioni, G. E., Karazsia, B. T., Chan, J., & Winton, A. S. W. (2016). Effectiveness of caregiver training in Mindfulness-Based Positive Behavior Support (MBPBS) vs. Training-as-Usual (TAU): A randomized controlled trial. FRONTIERS IN PSYCHOLOGY, 7. https://doi.org/doi:

Singh, N. N., Lancioni, G. E., Karazsia, B. T., Myers, R. E., Hwang, Y.-S., & Analayo, B. (2019). Effects of Mindfulness-Based Positive Behavior Support (MBPBS) Training Are Equally Beneficial for Mothers and Their Children With Autism Spectrum Disorder or With Intellectual Disabilities. FRONTIERS IN PSYCHOLOGY, 10, 385. https://doi.org/doi:https://dx.doi.org/10.3389/fpsyg.2019.00385

Singh, N. N., Lancioni, G. E., Medvedev, O. N., Hwang, Y.-S., & Myers, R. E. (2021). A Component Analysis of the Mindfulness-Based Positive Behavior Support (MBPBS) Program for Mindful Parenting by Mothers of Children with Autism Spectrum Disorder. Mindfulness, 12(2), 463-475. https://doi.org/doi:https://dx.doi.org/10.1007/s12671-020-01376-9

Son, C., Yoo, H. J., Kim, J. H., & Oh, M. (2023). Mood and Emotional Changes After PEERS® Program in Parents of Young Adults With Autism Spectrum Disorder [Article]. Journal of the Korean Academy of Child and Adolescent Psychiatry, 34(1), 30-36. https://doi.org/10.5765/jkacap.220023

Sood, D., Comer-HaGans, D., Barnec, A., Dowling, K., Kozy, K., Pranske, L., Redar, M., & Tietz, A. (2018). An Innovative Approach to Promote Health and Well-Being of Caregivers of Children With Autism Spectrum Disorder. OT Practice, 14-15. https://doi.org/doi:

Suzuki, M., Yamada, A., Watanabe, N., Akechi, T., Katsuki, F., Nishiyama, T., Imaeda, M., Miyachi, T., Otaki, K., Mitsuda, Y., Ota, A., & Furukawa, T. A. (2014). A failure to confirm the effectiveness of a brief group psychoeducational program for mothers of children with high-functioning pervasive developmental disorders: a randomized controlled pilot trial. Neuropsychiatric disease and treatment, 10, 1141-1153. https://doi.org/doi:https://dx.doi.org/10.2147/NDT.S60058

Swanson, S. E., Duijff, S. N., & Campbell, L. E. (2024). Care4Parents: An Evaluation of an Online Mindful Parenting Program for Caregivers of Children with 22q11.2 Deletion Syndrome [Article]. Advances in Neurodevelopmental Disorders. https://doi.org/10.1007/s41252-023-00389-4

Swarbrick, M., Fogerite, S. G., Spagnolo, A. B., & Nemec, P. B. (2021). Caregivers of People With Disabilities: A Program to Enhance Wellness Self-Care. Journal of psychosocial nursing and mental health services, 59(5), 25-32. https://doi.org/doi:https://dx.doi.org/10.3928/02793695-20210107-04

Tahmassian, K., Abadi, R. K., & Chimeh, N. (2012). The effectiveness of behavior management training on parental stress of autistic children's mothers. Developmental Psychology: Journal of Iranian Psychologists, 8(31), 269-278. https://doi.org/doi:

Tavakolizadeh, J., Dashti, S., & Panahi, M. (2012). The effect of rational-emotional training on mothers' mental health condition of children with mental retardation (Vol. 69). https://doi.org/doi:10.1016/j.sbspro.2012.11.457

Tendler, L. B. (2005). Parent-focused transition readiness program: Facilitating permanency planning for parents of adult children with mental retardation. Dissertation Abstracts International: Section B: The Sciences and Engineering, 65(10), 5425. https://doi.org/doi:

Thi Lan Anh, M., & Nujjaree, C. (2022). Effectiveness of a Family Management Intervention Program among Families of Children with Autism: A Randomized Controlled Trial. PACIFIC RIM INTERNATIONAL JOURNAL OF NURSING RESEARCH, 26(1), 63-76. https://doi.org/doi:

Tonge, B., Brereton, A., Kiomall, M., Mackinnon, A., King, N., & Rinehart, N. (2006). Effects on parental mental health of an education and skills training program for parents of young children with autism: a randomized controlled trial. JOURNAL OF THE AMERICAN ACADEMY OF CHILD AND ADOLESCENT PSYCHIATRY, 45(5), 561-569. https://doi.org/doi:https://dx.doi.org/10.1097/01.chi.0000205701.48324.26

Trelles, M. P., Katz, J., Sweeney, H., Foss-Feig, J., Mayo, L., & Nava-Palma, M. (2022). 3.56 A Family-Mediated Intervention to Improve Outcomes in Minority Children With ASD Affected by the COVID-19 Pandemic. JOURNAL OF THE AMERICAN ACADEMY OF CHILD AND ADOLESCENT PSYCHIATRY, 61(10), S246. https://doi.org/doi:https://dx.doi.org/10.1016/j.jaac.2022.09.335

Trustam, R. (2017). Building resilience in a changing world. Community Living, 31(2), 25-25. https://doi.org/doi:

Tumlu, C., & Akdogan, R. (2019). Looking at Life through a Different Window: Group Counselling for the Mothers of Disabled Children. INTERNATIONAL JOURNAL FOR THE ADVANCEMENT OF COUNSELLING, 41(2), 252-271. https://doi.org/doi:10.1007/s10447-019-09373-x

Tumlu, G. U., Akdogan, R., & Turkum, A. S. (2017). The Process of Group Counseling Based on Reality Therapy Applied to the Parents of Children with Disabilities. INTERNATIONAL JOURNAL OF EARLY CHILDHOOD SPECIAL EDUCATION, 9(2), 81-98. https://doi.org/doi:10.20489/intjecse.368465

Valizadeh, S., Davaji, R. B. O., & Dadkhah, A. (2009). The effectiveness of group coping skills training on reducing stress of mothers with disabled children. Iranian Rehabilitation Journal, 7(10), 9-12. https://doi.org/doi:

Vela, J. C., Ramos, N., & Perez, S. (2023). A pre-experimental evaluation of a 9-session training program with latinx parents of children with autism [Psychotherapy & Psychotherapeutic Counseling 3310]. Counseling Outcome Research and Evaluation, No-Specified. https://doi.org/https://dx.doi.org/10.1080/21501378.2023.2243324

Wahdan, M. M., Malak, M. Z., Al-Amer, R., Ayed, A., Russo, S., & Berte, D. Z. (2023). Effect of incredible years autism spectrum and language delays (IY-ASD) program on stress and behavioral management skills among parents of children with autism spectrum disorder in Palestine [Article]. Journal of Pediatric Nursing, 72, 45-52. https://doi.org/10.1016/j.pedn.2023.03.018

Wang, Q., Ng, S. M., & Zhou, X. (2023). The mechanism and effectiveness of mindfulness-based intervention for reducing the psychological distress of parents of children with autism spectrum disorder: A protocol of randomized control trial of ecological momentary intervention and assessment [Article]. PLoS ONE, 18(9 September). https://doi.org/10.1371/journal.pone.0291168

Worthington, R. C. (1992). Family support networks: Help for families of children with special needs. Family Medicine, 24(1), 41-44. https://doi.org/doi:

Yamanaka, T., Yuruki, K., Sanabe, Y., Yasutake, M., & Inoue, M. (2023). Assessing the Effectiveness of Real-Time Online Parent Training for Parents of Children with Diverse Neurodevelopmental Disorders Residing in the Community [Article]. Yonago Acta Medica, 66(4), 448-458. https://doi.org/10.33160/YAM.2023.11.010

Yap, D., Lau, L., Nasir, N., Cameron, C., Matthews, J., Tang, H. N., & Moore, D. W. (2014). Evaluation of a parenting program for children with behavioural problems: Signposts in Singapore. JOURNAL OF INTELLECTUAL & DEVELOPMENTAL DISABILITY, 39(2), 214-221. https://doi.org/doi:10.3109/13668250.2014.899567

Zaldo, C. S. (1996). A family systems model of service delivery for parents of children with autism: Promoting adaptation to family stress. Dissertation Abstracts International: Section B: The Sciences and Engineering, 57(2), 1429. https://doi.org/doi:

Zhao, M., You, Y., Chen, S., Li, L., Du, X., & Wang, Y. (2021). Effects of a Web-Based Parent-Child Physical Activity Program on Mental Health in Parents of Children with ASD. INTERNATIONAL JOURNAL OF ENVIRONMENTAL RESEARCH AND PUBLIC HEALTH, 18(24). https://doi.org/doi:https://dx.doi.org/10.3390/ijerph182412913

مژگان, غ., مریم, ا., محمود, گ., & امید, م. (2022). بررسی اثربخشی رویکرد پذیرش و عمل بر اساس طرح‌واره‌های ناسازگار اولیه با نقش واسطه‌ای دشواری‌های تنظیم هیجان بر ارتقاء کیفیت زندگی مادران کودکان استثنایی. Razi Journal of Medical Sciences, 29(4), 91-101. <https://doi.org/doi>:
