# Supplementary material for: Digital Psychological Wellbeing Interventions for Family Carers of Children and Adults With Intellectual and Developmental Disabilities: A Systematic Review
Source: J Appl Res Intellect Disabil. 2025 Jul 11;38(4):e70081. doi: 10.1111/jar.70081 (PMC12247015; doi:10.1111/jar.70081)
Supplement: Supplementary file 4 — Data S4. Supporting Information. [file JAR-38-e70081-s004.docx]

Table 3

Summary of participant feedback.

| **Source and origin** | **Data collection and analysis** | **Participant feedback** |
| --- | --- | --- |
| **Ahmed and Raj, 2023**  USA | **Tool**  Open and close-ended feedback questions  **When?**  After every module  **Analysis method**  Not reported | - Would recommend to a friend - 92.9% for first module, 97.6% for the second module, 95.5% for the third module and 92.7% for the fourth module. - Easy or very easy to find time to engage – average 61%. - Difficult or very difficult to find time to engage – average 22%. - Intervention activities relaxing or very relaxing - 52.6%. - Intervention activities stressful or very stressful - 9.4%. - Aspects found helpful: “learning that self-compassion is always available to me,” “helps me to remember that to be imperfect is to be human,” and “considering my own health”. - Suggestions: incorporate the intervention as part of child’s appointments, offer a brief format of the intervention, provide additional resources on specific topics, include more examples and add self-worth quotes. |
| **Bekhet, 2017a**  USA | **Tool**  Weekly homework recordings and  open ended questions  **When?**  After every session  **Analysis method**  Responses were coded based on the use of eight positive thinking skills. | - Most used skills: interruption of pessimistic thoughts (97%), transforming negative thoughts (93%), and controlling negative thoughts (93%). - Able to learn all parts of the intervention – 100%. - Wanted more time – 11%. - Suggestions: include transcripts, printouts, written material and more examples, more interactions and feedback, allow more time. |
| **Bekhet, 2017b**  USA | **Tool**  Open-ended questions  **When?**  Post-test  **Analysis method**  Content analysis | - Easiest parts of the intervention: PowerPoints and visualization (39%), breaking down problems (14%) - Most challenging parts of the intervention: remembering to use the strategies (25%), finding time (11%), breaking down problems (7%). - Most interesting: whole intervention (50%), turning negative thoughts into positive (32%), relaxation (14%), examples (11%), breaking down problems (4%). - Least interesting: relaxation (11%), first week (7%). - Reported a need for the intervention – 82%. - Other carers need the intervention – 89%. |
| **Clifford and Minnes,**  **2013**  Canada | **Tool**  Post-session surveys, post-treatment questionnaire  **When?**  After every session and at post-test  **Data analysis**  Not reported | - Overall support groups were rated as useful. - The most useful: opportunity to connect (40%), information about resources (25%), facilitation of the group (15%), helping with research (5%). - The least useful: technical issues (15%), differences in experiences (needs or location) (15%), attendance (10%), difficult topics (10%), others’ complaints (5%), wanted more resources from the facilitator (5%). |
| **Curl and Hampton,**  **2023**  USA | **Tool**  Focus groups and one interview  **When?**  Post-test  **Data analysis**  Thematic analysis | - Participants agreed that the virtual delivery and group format were beneficial. - Challenges: chat feature not working; sharing tables and resources in a virtual format. - Four themes identified:   - Self-care – Participants signed up for the study because they were critical of themselves. They stated that the workshop helped increase their self-care.   - Stress – Participants reported high levels of stress in their lives and challenges related to parenting an autistic child. Intervention helped with their self-patience and self-kindness.   - Interpersonal connections – Participants reported feeling more connected to their family after the intervention. They appreciated sharing experiences with other participants.   - Barriers – Participants reported difficulties practicing mindfulness around their children and prioritising time for self-care. Participants reported that travel time and childcare are barriers to in person meetings. They also identified lack of connection with other participants and technical difficulties as barriers to online meetings. |
| **Fenning et al., 2023**  USA | **Tool**  The Parent Satisfaction Questionnaire (McIntyre, 2008)  **When?**  Post-test  **Data analysis**  Not reported | Feedback of participants from virtual and in-person groups:   - Would recommend or strongly recommend the intervention to others – 95%. - Positive or very positive feelings about the intervention – 93%. - Improvements in areas that led them to sign up for the study – 79%; . - Easy or very easy elements: information presented (81%), meditations (71%), group discussions (81%), skills at home (62%), homework (57%). - Useful or extremely useful elements: information presented (90%), meditations (88%), group discussions (95%), skills at home (83%), homework (78%).   Feedback of participants from online groups only:   - Positive or very positive feelings about the intervention – 85%. - Online format beneficial – 95%. - Elements of virtual delivery that were endorsed: being at home (74%), not having to commute (68%), interact with others online (79%), engagement (58%), group discussions (68%), option to turn off video or mute (89%), partner participation (11%). - Challenges to online participation: lack of in-person interactions (43%), juggling demands (57%), limited privacy (43%), technical issues (32%). - Prefer online delivery format – 45%. - Willing to try online format again in the future – 70%. - Increased confidence with accessing online clinical services – 64%.   Virtual vs. in-person groups:   - High levels of satisfaction regardless of delivery mode (virtual or in-person), but participants in the virtual groups reported more ease in using learnt skills at home (83% vs. 37%) and in completing homework (74% vs. 37%). |
| **Flynn et al., 2020**  UK | **Tool**  Semi-structured telephone interviews  **When?**  After intervention period finished – exact timeline not reported  **Data analysis**  Deductive framework analysis approach (Ritchie and Spencer 1994) | Motivation to participate   - Reduce stress levels or improve well-being (n = 12). - Contribute to research (n = 8).   Acceptability   - Participants were happy with the randomization - Be Mindful+ - phone calls were motivating, provided reassurance and time to reflect. - Be Mindful+ - did not feel they would not be able to complete the online intervention without the phone calls. - Be Mindful – appreciated being able to complete the intervention in their pace. - Be Mindful – did not have time for the phone calls. - Be Mindful – did not feel having phone calls would change the outcome for them. |
| **Kangavary et al., 2023**  USA  Case study | **Tool**  The Program Feedback Scale (PFS; Schleider et al., 2020)  **When?**  At post-test  **Data analysis**  Not reported | - Session 1 – Participant reported positive experiences of the session and would recommend it to a friend. They appreciated being able to connect with other carers. - Session 2 – Participant reported not enjoying this session and recommend changes to session duration and meditation activities. - Session 3 – Participant reported not enjoying this session but agreeing with the overarching message. They stated they would prefer to not have homework and meet in-person rather than online. - Session 4 – Participant reported enjoying the session activity but not finding it easy. They stated they would recommend this session to a friend and found elements of the group and covered content helpful. |
| **Kuhlthau et al., 2020**  USA | **Tool**  Open-ended questions in the feedback survey  **When?**  3 months after enrolment  **Data analysis**  Not reported | - Sessions were the right length – 73%. - Number of sessions was enough – 70%. - Virtual intervention was “quite helpful”. - Practice of intervention techniques was useful. - Connecting with others was useful. - Some said that they wanted a shorter program and others wanted a longer one. |
| **Kulbas and Ozabaci,**  **2022**  Turkey | **Tool**  Semi-structured interview  **When?**  At post-test  **Data analysis**  Content analysis | Identified themes were:   - Learning how to cope with - Recognizing he sources of support - Positive perception-acceptance - Empathy and Development |
| **Lake et al.,**  **2022**  Canada | **Tool**  Open-ended survey questions  **When?**  After each session and at post-test  **Data analysis**  Not reported | - Mean weekly satisfaction - 4.15 to 4.46 (out of 5). - Over 80% of participants agreed or strongly agreed with questions related to structure and delivery, curriculum and content and relevance of the course:   - Relating to other families - 93%.   - Easy to understand - 95%.   - Interesting - 92%.   - Co-facilitation by a family carer – 59%. - Expectations of the intervention were met – 93%. - Suggested improvements: having a recording of the session, printed materials before the session, longer sessions, sessions at different times of day, more breakout sessions, grouping participants based on region or diagnosis of their family member with IDD, and more co-facilitators. - Reported change after the intervention – 78%. - Learnt strategies helpful or somewhat helpful – 80%. |
| **Lunsky et al., 2021**  Canada | **Tool**  Open-ended questions about intervention satisfaction  **When?**  At post-test  **Data analysis**  Not reported | - Satisfaction with the intervention – 4.75 out of 5 - was comparable to in-person delivery. - Satisfaction with technology and virtual format – 5.73 out of 7. - Most felt connected to others during the intervention. - Three themes were identified:   - The value of connecting with others in similar situations.   - The benefits of and challenges with technology.   - Utility of mindfulness-based skills. - Helpful aspects – strategies/skills learnt, other participants and facilitators. - Suggestions for changes - automatic reminders, recordings of sessions, support for the online forum and visible agenda during the session. |
| **Luberto et al., 2021**  USA  We are only reporting data from the autism group. | **Tool**  Closed – and open-ended questions about the SMART-3RP programme  **When?**  Within 2 weeks of completing the intervention  **Data analysis**  Content analysis | - Intervention had the right number of sessions - 70% - Sessions were of the right length - 73% - Group structure was right - 78% - Comfortable during the group sessions - 84% - Practicing relaxation at home was helpful - 87% - Learnt skills were helpful - 81% - Practiced relaxation at home - daily 24%, few times a week 54%, and once or twice a week 16%. - Mean scores for session helpfulness - 1.59 (SD=0.90) to 1.95 (SD=.97) (out of 5).   Qualitative feedback   - Most reported positive experiences of virtual delivery. - Virtual delivery saves time and money and enables access. - Virtual delivery was less personal but better than not participating. - Some had technology issues and found video distracting. - Helpful aspects: social support, learnt skills, time to focus on themselves, structure and delivery of the intervention. - Challenging aspects: irrelevant/invalidating content, homework, study procedures, structure of the intervention and participation of others, session length, lack of flexibility, and virtual distractions. - Suggested improvements: materials sent in advance, ensuring group cohesion, longer intervention, less structure, consistent schedule, more reliable online platform, clear expectations, and including content relevant to their experience. |
| **Osborn**  **2020**  (unpublished thesis)  Australia | **Tool**  Net Promoter Scores (NPS)  **When?**  At post-test  **Data analysis**  Not reported | - The total Net Promoter Score (NPS) was 30, indicating generally positive perception of the intervention. |
| **Padgett**  **2020**  (unpublished thesis)  USA | **Tool**  Evaluation  of the Mindful Parenting Course and Personal Change **When?**  At post-test  **Data analysis**  Not reported | - Intervention was of value and led to positive changes – 100%. - Made changes to their family life after the intervention – 100%. - Felt more mindful in parenting – 100%. - Planned to continue with learnt strategies – 100%. - Intervention was sufficient – 90%. - Completed meditation – 1-2 times 30%, 3-4 times 30% and 5-7 times 40%. - Spending time with their child more mindfully – 70%. - Most noticed some positive changes after the intervention. - Would like more training in: parenting 30%, managing their difficulties 40%, their child difficulties 40%, relationship with partner 40%, and family difficulties 30%. - Most reported that the intervention was important to them. |
| **Tilson,**  **2022**  (unpublished thesis)  USA | **Tool**  Social Validity Questionnaire  **When?**  At post-test  **Data analysis**  Not reported | Strongly agreed:   - Videos were informative and helpful – 100%. - Videos led to change in behaviour – 80%. - Videos were engaging – 80%. - Intervention content was structured well – 80%. - Resources were accessible – 60%. - Understand how to use learnt strategies – 80%. - Exercises led to positive outcomes for me – 100%. - Exercises led to positive changes for child and family – 40%. - Would recommend learnt strategies – 100%. - Would recommend the intervention – 100%. - Beneficial aspects: strategies and tools, exercises, validation, small changes. - Suggested changes: clear expectations and homework, easier data collection method, more discussions. |
| **Zhou**  **2022**  (unpublished thesis)  USA | **Tool**  Questionnaire  **When?**  At post-test  **Data analysis**  Not reported | Results not reported. |
| **Zimmerman**  **2013**  (unpublished thesis)  USA | **Tool**  Open-ended questionnaire questions  **When?**  At pre- and post-test  **Data analysis**  Thematic analysis | Identified categories:   - Parenting ability: Some felt more confident, while others felt they did not learn enough. - Social wellbeing: Feelings of isolation, stress and depression have not improved for most, but they felt more connected. - Web-based groups: All reported experiencing technical issues, but they would take part in virtual intervention again in the future. They noted convenience of virtual format. |
