# Supplementary material for: Digital Psychological Wellbeing Interventions for Family Carers of Children and Adults With Intellectual and Developmental Disabilities: A Systematic Review
Source: J Appl Res Intellect Disabil. 2025 Jul 11;38(4):e70081. doi: 10.1111/jar.70081 (PMC12247015; doi:10.1111/jar.70081)
Supplement: Supplementary file 5 — Data S5. Supporting Information. [file JAR-38-e70081-s001.docx]

Table 1

Summary of quality appraisal scores based on Mixed Method Appraisal Tool (MMAT; Hong et al., 2018) for studies employing quantitative descriptive design.

|  | **Ahmed and Raj, 2023** | **Bourke-Taylor et al., 2022** | **Flujas-Contreras et al., 2021** | **Kangavary et al., 2023** | **Lake et al., 2022** | **Luberto et al., 2021** | **Lunsky et al., 2021** | **Osborn,**  **2020** | **Tilson,**  **2022** |
| --- | --- | --- | --- | --- | --- | --- | --- | --- | --- |
| S1. Are there clear research questions? | Yes | Yes | Yes | No | Yes | Yes | Yes | No | Yes |
| S2. Do the collected data allow to address the research questions? | No | Yes | Yes | Can’t tell | Yes | Yes | Yes | Yes | Yes |
| 4.1. Is the sampling strategy relevant to address the research question? | Yes | Yes | Yes | Yes | Yes | Yes | Yes | Yes | Yes |
| 4.2. Is the sample representative of the target population? | No | Yes | No | No | Yes | No | No | No | No |
| 4.3. Are the measurements appropriate? | Yes | Yes | Yes | Yes | Yes | Yes | Yes | Yes | Yes |
| 4.4. Is the risk of nonresponse bias low? | Yes | No | Yes | Yes | Yes | Yes | No | No | Yes |
| 4.5. Is the statistical analysis appropriate to answer the research question? | No | Yes | Yes | No | Yes | Yes | Yes | Yes | Yes |
| Quality indicators met | **4/7** | **6/7** | **6/7** | **3/7** | **7/7** | **6/7** | **5/7** | **4/7** | **6/7** |

Table 2

Summary of quality appraisal scores based on Mixed Method Appraisal Tool (MMAT; Hong et al., 2018) for studies employing quantitative randomised controlled trial design.

|  | **Bekhet, 2017a** | **Bekhet, 2017b** | **Fenning et al., 2023** | **Hemdi and Daley, 2017** | **Kuhlthau et al., 2020** | **Padgett,**  **2020** | **Pandya,**  **2020** | **Whitney and Smith, 2014** | **Zhou,**  **2022** |
| --- | --- | --- | --- | --- | --- | --- | --- | --- | --- |
| S1. Are there clear research questions? | Yes | Yes | No | Yes | Yes | Yes | Yes | Yes | Yes |
| S2. Do the collected data allow to address the research questions? | Yes | Yes | Can’t tell | Yes | Yes | Yes | Yes | Yes | Yes |
| 2.1. Is randomization appropriately performed? | Can’t tell | Can’t tell | Can’t tell | No | Yes | No | Yes | Yes | Yes |
| 2.2. Are the groups comparable at baseline? | Can’t tell | Can’t tell | Yes | Yes | Yes | Can’t tell | Yes | Can’t tell | Yes |
| 2.3. Are there complete outcome data? | Yes | Yes | Yes | Yes | No | No | Yes | No | No |
| 2.4. Are outcome assessors blinded to the intervention provided? | Can’t tell | Can’t tell | Can’t tell | Yes | Can’t tell | Can’t tell | No | Can’t tell | Can’t tell |
| 2.5 Did the participants adhere to the assigned intervention? | No | No | Yes | Yes | No | No | No | No | No |
| Quality indicators met | **3/7** | **3/7** | **3/7** | **6/7** | **4/7** | **2/7** | **5/7** | **3/7** | **4/7** |

Table 3

Summary of quality appraisal scores based on Mixed Method Appraisal Tool (MMAT; Hong et al., 2018) for studies employing mixed-method design.

|  | **Curl and Hampton,**  **2023** | **Flynn et al., 2020** | **Kulbas and Ozabaci,**  **2022** | **Zimmerman,**  **2013** |
| --- | --- | --- | --- | --- |
| S1. Are there clear research questions? | Yes | Yes | Yes | Yes |
| S2. Do the collected data allow to address the research questions? | Yes | Yes | Yes | Yes |
| 5.1. Is there an adequate rationale for using a mixed methods design to address the research question? | Yes | Yes | Yes | Yes |
| 5.2. Are the different components of the study effectively integrated to answer the research question? | Yes | Yes | Yes | Yes |
| 5.3. Are the outputs of the integration of qualitative and quantitative components adequately interpreted? | Yes | Yes | Yes | Yes |
| 5.4. Are divergences and inconsistencies between quantitative and qualitative results adequately addressed? | Yes | Yes | Yes | Yes |
| 5.5. Do the different components of the study adhere to the quality criteria of each tradition of the methods involved? | Yes | Yes | Yes | Yes |
| Criteria met | **7/7** | **7/7** | **7/7** | **7/7** |

Table 4

Summary of quality appraisal scores based on Mixed Method Appraisal Tool (MMAT; Hong et al., 2018) for studies employing quantitative non-randomised design.

|  | **Clifford and Minnes,**  **2013** |
| --- | --- |
| S1. Are there clear research questions? | Yes |
| S2. Do the collected data allow to address the research questions? | Yes |
| 5.1. Is there an adequate rationale for using a mixed methods design to address the research question? | No |
| 5.2. Are the different components of the study effectively integrated to answer the research question? | Yes |
| 5.3. Are the outputs of the integration of qualitative and quantitative components adequately interpreted? | No |
| 5.4. Are divergences and inconsistencies between quantitative and qualitative results adequately addressed? | Yes |
| 5.5. Do the different components of the study adhere to the quality criteria of each tradition of the methods involved? | Yes |
| Criteria met | **5/7** |
